# Multimuscle Surface-EMG Characterization of Upper-Limb Fatigue During Repetitive Haptic Interaction for Health 5.0 Applications

**DOI:** 10.3390/bioengineering13090982

**Published:** 2026-08-26

**Authors:** Mohammad Alja’afreh, Nasser Mustafa, Ali Karime

**Affiliations:** 1 Department of Communications Engineering, King Abdullah II School of Engineering, Princess Sumaya University for Technology, Amman 11941, Jordan; 2 Department of Computer Science, College of Science, Mathematics and Technology, Wenzhou-Kean University, Wenzhou 325060, China; 3 Department of Electrical and Computer Engineering, Royal Military College of Canada, Kingston, ON K7K 7B4, Canada; ali.karime@rmc-cmr.ca

**Keywords:** surface electromyography, muscle fatigue, haptic interaction, medical robotics, robotic surgery, robot-assisted rehabilitation, virtual reality, Health 5.0, Industry 5.0, biomedical signal processing

## Abstract

Health 5.0 increasingly involves medical robots and haptic systems that sustain physical interaction with patients and clinicians. This proof-of-concept study examined fatigue-related surface electromyography (sEMG) spectral changes during a 400 s repetitive haptic-writing task in 20 adults. Five upper-limb muscles were monitored at 1000 Hz, and mean frequency (MNF) and median frequency (MDF) trajectories were summarized by fitted start-to-end spectral decline and combined into arm-level indices. MDF had the higher association with the archived participant-level fatigue-analysis score (r=0.954 versus r=0.783; Δr=0.171; Holm-adjusted p=0.0035). Small-sample influence analysis supported the stability of this within-sample ordering: after omitting each participant in turn, Δr remained positive in 20/20 analyses (range 0.125–0.218), although the exact paired label-swap sensitivity test remained inconclusive (p=0.082). Exploratory leave-one-participant-out calibration produced lower held-out error for MDF (MAE 4.38, RMSE 5.87 percentage points) than for MNF (MAE 9.24, RMSE 12.02 percentage points). The raw seven-category Q2 responses and the archived 0–100 analysis score are reported as separate data products because they are not numerically equivalent under direct linear rescaling. Accordingly, the results identify MDF as the more promising spectral summary for prospective validation in this task, rather than establishing universal superiority or numerical replacement of subjective fatigue. The experiment was a laboratory haptic-writing study and did not test a surgical robot, rehabilitation robot, patient population, or clinical controller; Health 5.0 is therefore presented as a translational motivation rather than a demonstrated application.

## 1. Introduction

Haptic interfaces extend virtual and remote interaction by coupling a user’s motor actions with tactile or kinesthetic feedback [1]. They are increasingly investigated for virtual-reality training and collaborative interaction [2,3], rehabilitation [4,5,6], teleoperation, and medical robotics [1,7,8]. In Health 5.0 applications, haptic devices may connect surgeons to master–slave instruments, patients to rehabilitation end effectors, or clinicians to shared-control therapeutic robots [4,5,6]. A contemporary haptic communication system may integrate motion and force sensing, mechanical or electrical feedback, real-time communication, and adaptive software within a closed perception–action loop [1]. Recent developments illustrate the increasing duration and physical intensity of such interactions. Wearable systems have harvested energy from repeated elbow movements to sustain untethered haptic feedback [9], while shared haptic cues have supported rapid, repetitive turn-taking between remote collaborators in virtual reality [3]. These developments broaden the practical scope of haptic interaction, while physiological demand becomes a parallel design consideration for the human operator or patient.

Industry 5.0 complements the technology-centered focus of Industry 4.0 by emphasizing sustainability, resilience, and human centricity, including the well-being of workers within advanced production systems [10,11]. Human-centric manufacturing increasingly combines operator expertise with collaborative robots, immersive training, teleoperation, and adaptive cyber–physical systems [12]. In these settings, haptic feedback can extend perception, manipulation, and remote control, but repetitive kinesthetic interaction may also increase cumulative upper-limb loading. This places unobtrusive characterization of the operator’s physical state among the bioengineering challenges associated with comfort, precision, and safe performance. Repetitive haptic writing was used as a controlled model of the sustained force–feedback interactions relevant to human-centered Industry 5.0 work systems.

Health 5.0, often discussed as Healthcare 5.0, applies a related human-centric principle to digitally supported care, rehabilitation, prevention, and medical robotics. It emphasizes personalized services, continuous physiological awareness, and collaboration between people and intelligent systems rather than the replacement of clinical judgment [13,14]. Wearable physiological sensors are central to this transition because they can extend assessment beyond isolated clinical encounters and provide longitudinal information on movement and neuromuscular function [15]. Haptic medical robots are relevant in two complementary settings. In robot-assisted surgery and telemanipulation, the clinician repeatedly controls a master console while maintaining visual attention and stable upper-limb posture. Robotic platforms can reduce some ergonomic demands relative to conventional laparoscopy, but objective sEMG studies show that muscle-specific activation and localized fatigue remain and can differ across the trapezius, deltoid, forearm, and upper-arm muscles [7,8]. In robot-assisted rehabilitation, the patient repeatedly exchanges force with an end effector or exoskeleton; assistance may reduce fatigue, whereas excessive resistance or repetition may alter movement quality and the relation between EMG and interaction force [4]. Recent EMG-driven rehabilitation protocols have therefore incorporated fatigue checks based on median frequency and root-mean-square (RMS) amplitude [5,6].

**Figure 1:**
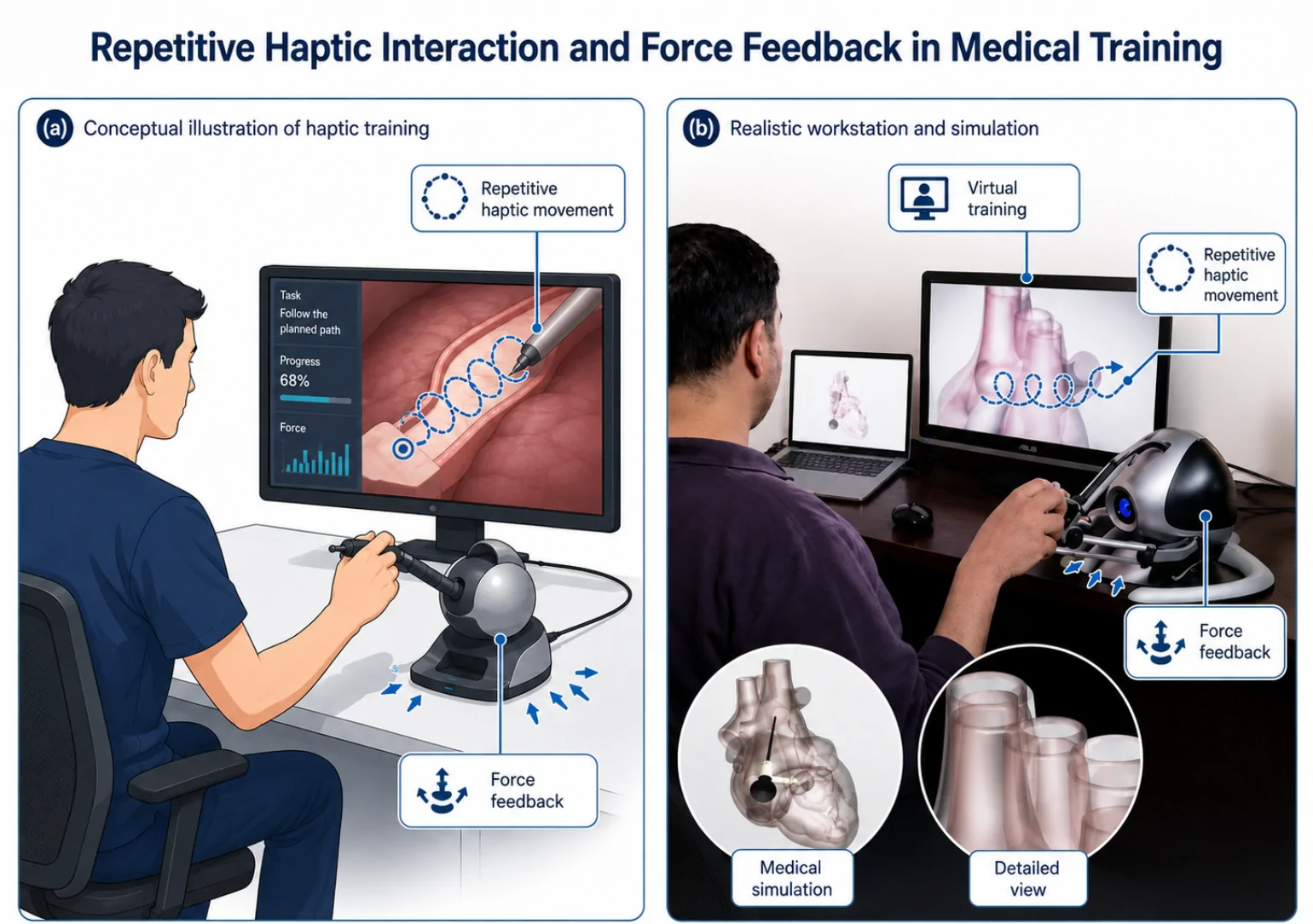
Panel (**a**) presents a conceptual illustration of a haptic training task in which a user repeatedly guides a stylus along a simulated procedural path while receiving force feedback. Panel (**b**) presents a realistic workstation and simulation environment that reflects the same interaction principles. Together, the panels emphasize repetitive haptic movement and force feedback as central characteristics of Health 5.0 training systems and as potential sources of cumulative upper-limb loading.

This motivation is illustrated in Figure 1, which groups a conceptual view of haptic medical training with a realistic workstation example. In both views, the user performs repetitive stylus movements while receiving force feedback from the interface, highlighting the sustained perception–action loop that can contribute to cumulative upper-limb loading in Health 5.0 applications.

These observations define fatigue-aware medical robotics as a distinct Health 5.0 design objective. In clinician-operated systems, the relevant physiological concern is prolonged static and repetitive loading without procedural distraction. In rehabilitation systems, the corresponding concern is excessive exercise-induced fatigue relative to the intended therapeutic challenge. Accordingly, repetitive haptic exposure provides a useful bioengineering model for characterizing upper-limb physiological demand during sustained interaction.

Sustained or repetitive manipulation of a force–feedback interface can require continuous activation of several muscles spanning the wrist, elbow, and shoulder. Over time, this loading may reduce the capacity of the neuromuscular system to maintain the required force or movement and may affect movement accuracy, task performance, comfort, and perceived quality of experience. Repeated exposure is also relevant to cumulative mechanisms associated with work-related musculoskeletal disorders [16]. Earlier research examined fatigue in simulator-based tasks through psychomotor and cognitive performance [17], modeled fatigue-related changes in muscle force production [18], and evaluated physical demands during maintenance, assembly, and computer-input activities [19,20]. However, the physiological demands of dynamic haptic–virtual-reality interaction remain less thoroughly characterized than the technical performance, realism, or communication requirements of the haptic system itself.

Fatigue in virtual environments encompasses distinct physiological and psychological processes. Sensory conflict, sustained attention, and cognitive workload may produce cognitive fatigue, whereas repeated force production and movement may produce local neuromuscular fatigue. Recent work on virtual-reality remapping has shown that visual–vestibular, visual–proprioceptive, and visual–experiential conflicts can influence the onset and severity of cognitive fatigue [21]. The present work focuses on task-induced fatigue of the upper-limb musculature associated with repetitive interaction through a force–feedback stylus. Subjective tiredness, cognitive fatigue, clinical fatigue, and reduced neuromuscular function represent distinct constructs with different measurement requirements.

Subjective ratings provide important information about perceived effort and discomfort, but they are difficult to use as the sole basis for continuous or real-time adaptation. Ratings are influenced by individual interpretation, expectations, previous experience, and the time at which they are collected. Haptic-system evaluation therefore increasingly requires objective measures that can complement participant reports [1]. Surface electromyography (sEMG) offers a non-invasive approach for monitoring muscle activation during human–machine interaction. During sustained or repeated contractions, changes in motor-unit recruitment, conduction velocity, synchronization, and metabolic state are commonly accompanied by a redistribution of the sEMG power spectrum toward lower frequencies [22,23]. Mean frequency (MNF) and median frequency (MDF) are consequently among the most frequently used spectral indicators of localized muscle fatigue [24].

MNF represents the power-weighted average frequency of the sEMG spectrum, whereas MDF divides the spectrum into two regions containing equal power. Both measures generally decrease as fatigue develops, although their behavior may also be affected by contraction intensity, muscle geometry, movement, electrode placement, and signal contamination. MDF is often considered less sensitive than MNF to extreme spectral values and broadband disturbances, but the relative performance of these measures depends on the task and analysis procedure. Previous wearable systems have used frequency-domain sEMG features to track fatigue during body movement [25]. More recently, frequency-domain measures have also been used to evaluate recovery after force–electrical haptic stimulation [26]. This motivates evaluation of whether MNF and MDF can provide meaningful subject-level estimates of fatigue during a dynamic, multimuscle haptic task rather than during a predominantly static or highly constrained contraction.

This multimuscle perspective is important because upper-limb haptic interaction is not generated by a single muscle. Wrist positioning, stylus control, elbow movement, and shoulder stabilization distribute the task demand across several muscles, and their relative contributions may change during the interaction. A single-muscle measure may therefore fail to represent the fatigue experience of the complete limb. In addition, much of the haptic literature has emphasized actuator performance, network behavior, immersion, energy consumption, or task coordination, with comparatively less attention to objective estimation of the user’s evolving neuromuscular state. A multimuscle fatigue indicator represents a potential physiological input for closed-loop modulation of force–feedback magnitude, workspace, interaction gain, task difficulty, or rest timing.

This study is motivated by a shared design problem across Health 5.0 medical robotics and Industry 5.0: haptic systems are intended to augment human capability, yet the same repeated force–feedback interaction can progressively load the surgeon, therapist, patient, or industrial operator. In surgical teleoperation, fatigue may affect console posture, hand control, and sustained precision; in robotic rehabilitation, it may affect the patient’s ability to complete repetitions with the intended movement quality. An interface can therefore remain technically accurate and subjectively engaging while becoming physically unsustainable. Physiological feedback offers one mechanism for individualized modulation of assistance, resistance, haptic gain, task pace, or rest timing. The present work evaluates an initial sensing question for such systems—whether multimuscle sEMG spectral summaries relate to perceived arm fatigue during a controlled dynamic task—rather than proposing a ready-to-deploy medical or industrial controller.

Recent work has expanded both the database and the application landscape for sEMG-based fatigue monitoring. Dynamic upper-limb datasets now pair multichannel sEMG with self-perceived fatigue across several movements [27], recent reviews emphasize that wearable upper-limb fatigue indices remain sensitive to task definition and feature directionality [28], and rehabilitation systems increasingly use EMG-derived fatigue estimates inside adaptive control loops [5,6]. Comparative algorithm studies further show that fatigue-index performance is task dependent rather than universally fixed [29]. The novelty of the present work is therefore not the isolated use of MNF or MDF but an integrated haptic-task framework that: (i) characterizes representative five-muscle spectral trajectories during a 400 s dynamic exposure; (ii) derives transparent MNF- and MDF-based arm indices from all five monitored muscles; (iii) compares the indices with dependent-correlation inference, exact/randomization sensitivity, leave-one-participant-out influence analysis, uncalibrated scale-correspondence analysis, and held-out internal calibration; and (iv) translates the resulting evidence into a prospective Health 5.0/Industry 5.0 validation pathway. The raw Q2 questionnaire distribution and the archived continuous analysis criterion are explicitly treated as separate numerical data products.

Recent human–robot collaboration research reinforces two methodological points that are directly relevant here. Chand et al. quantified fatigue during repetitive dynamic human–robot operations using sEMG together with task-load information, emphasizing personalized fatigue profiles rather than assuming a single universal muscle response [30]. The earlier peer-reviewed report of the present haptic paradigm documented the Geomagic/Delsys setup, 1 kHz sEMG acquisition, Bessel filtering, a 400 s exposure, and 0.125 s windows with 0.0625 s overlap [31]. We use that report only to recover settings that were explicitly documented; parameters it did not state are not inferred retrospectively.

Accordingly, the present study investigates upper-limb muscular fatigue during a repetitive haptic-writing task in virtual reality. Surface EMG and triaxial acceleration were recorded from the flexor carpi radialis, brachioradialis, triceps brachii, biceps brachii, and deltoid while participants repeatedly manipulated a force–feedback stylus. The study had three objectives: (1) to characterize representative temporal MNF/MDF behavior during dynamic haptic interaction; (2) to derive participant-level multimuscle arm indices from the five monitored muscles; and (3) to compare the MNF- and MDF-derived arm indices on the archived participant-level analysis scale using association, participant influence, internal calibration, and uncalibrated scale correspondence, while reporting raw post-task Q2 responses separately. The relative performance of the two indices was compared without assuming a priori superiority. The broader aim is to evaluate whether frequency-domain sEMG analysis can support future fatigue-aware, human-centric haptic interfaces relevant to Industry 5.0 and Health 5.0.

The remainder of this paper is organized as follows. Section 2 reviews objective sEMG fatigue assessment, haptic workload, and fatigue monitoring in Health 5.0 medical robotics and virtual environments. Section 3 describes the participants, experimental platform, signal-processing pipeline, multimuscle index construction, questionnaire and archived-score provenance, and statistical analyses. Section 4 presents the task characteristics, representative physiological signals, participant-level indices, association, exploratory scale-correspondence and internal-calibration analyses, and questionnaire outcomes. Section 5 discusses the physiological interpretation, methodological strengths, limitations, future research, and implications for fatigue-aware Health 5.0 medical robots and Industry 5.0 systems. Section 6 summarizes the principal conclusions.

## 2. Related Work

### 2.1. Objective Assessment of Neuromuscular Fatigue

Neuromuscular fatigue is commonly described as a task-dependent reduction in the capacity to generate the force or power required for a prescribed activity. It can arise from interacting central and peripheral mechanisms, and its manifestation depends on contraction intensity, duration, muscle group, movement strategy, and the performance criterion used to define task failure [32]. Consequently, no single physiological variable constitutes a universal measure of fatigue. Direct performance outcomes, such as a decline in maximal voluntary force, endurance time, or task accuracy, remain important reference measures, whereas physiological signals are used to characterize mechanisms or provide continuous estimates when repeated maximal testing is impractical [23].

Surface electromyography is particularly attractive for human–machine and ergonomic studies because it can be recorded non-invasively and continuously during task performance. Time-domain measures, including average rectified value and root-mean-square amplitude, reflect the combined effects of motor-unit recruitment, discharge behavior, action-potential shape, and electrode geometry. During a sustained submaximal contraction, amplitude often increases as additional motor units are recruited to maintain force; however, this response is not monotonic across all tasks and may plateau or decline near task failure. Amplitude measures are also strongly affected by contraction level, changes in limb posture, movement of the electrodes relative to the muscle fibers, subcutaneous tissue, and cancellation between motor-unit action potentials [23,33]. They are therefore useful indicators of activation demand but do not constitute fatigue measures without accounting for force and task mechanics.

Frequency-domain analysis is based on the well-established observation that the sEMG spectrum commonly shifts toward lower frequencies during fatiguing contractions. Decreased muscle fibre conduction velocity, changes in intracellular pH and membrane excitability, and motor unit behaviour modification are all responsible for such spectral compression but with differing relative contributions depending on the type of contraction and muscle [22,23]. The mean frequency is a power-weighted average of the spectrum, while the median frequency separates the spectrum into two regions with equal power. Both of these have been extensively used as indices of the time evolution of local fatigue [24]. The median frequency is less sensitive to the presence of extreme values in the spectrum, while the mean frequency retains the information from the full spectrum; neither is superior in all circumstances.

Interpreting spectral indices is easier in sustained isometric contractions since the signal may be considered quasi-stationary within short analysis windows. The dynamics of the task introduce additional complications due to changes in muscle length, contraction forces, fibre alignment, and electrode–muscle positioning. Movement artifacts and alternating active and non-active phases may also affect spectral estimates during dynamic tasks. Studies reviewing the dependence of the contraction level and spectral parameters confirm that the mean and median frequencies may depend on force [33]. In the current paper, mean and median neural frequencies (MNFs and MDFs) were used as descriptors of spectral changes associated with fatigue during continuous haptic interaction. Such an interpretation is based on persistent, long-horizon changes across multiple repetitions of the movement cycle, estimated from short, overlapping windows and low-order trajectories. Force, position, and acceleration signals were measured in parallel to provide a mechanical context to the sEMG trajectories.

Earlier wearable systems demonstrated the feasibility of using declining sEMG frequency features to track fatigue during body movement [25]. Related ergonomic studies quantified upper-limb muscle loading during pen-tablet interaction [20], while simulation-based approaches estimated fatigue during maintenance and assembly operations [19]. These studies established that objective monitoring can complement subjective reports, but they also leave unresolved how to combine signals from several muscles during a dynamic haptic task. The present work addresses this problem by evaluating subject-level indices derived from five upper-limb muscles rather than treating a single muscle or a single representative trial as a proxy for the fatigue state of the entire arm.

### 2.2. Fatigue in Haptic and Virtual Environments

Research on fatigue in haptic and virtual environments has developed along several partly separate lines. Early work evaluated the consequences of operator fatigue through task completion, psychomotor performance, or cognitive performance in simulation-based settings [17]. Other studies modeled fatigue-related reductions in muscle force [18] or estimated physical demand in virtual maintenance and assembly tasks [19]. Experiment-based approaches subsequently introduced wearable sEMG systems for continuous fatigue tracking [25]. Collectively, these studies established the relevance of fatigue to simulated and technology-mediated work, but most did not examine coordinated activity across the wrist, elbow, and shoulder during force–feedback interaction.

Contemporary haptic systems create stronger motivation for user-state monitoring because they support longer, more repetitive, and more physically coupled experiences. A recent survey identified wearability, power supply, multimodal integration, reliability, and evaluation as major challenges for haptic communication systems [1]. Teng et al. demonstrated a batteryless wearable interface that harvested energy from repeated elbow movement and concealed the resulting resistance within contextually appropriate virtual actions, such as swimming or rowing [9]. The approach extends the duration of untethered haptic experiences, but it also illustrates how energy-management strategies can deliberately transfer mechanical demand to the user. In remote collaborative VR, Jang et al. studied short-cycle repetitive turn-taking under four haptic-feedback conditions and combined behavioral, ECG, EMG-amplitude, and questionnaire measures [3]. Bidirectional haptic feedback improved action-timing synchrony and reduced perceived visual dependence, yet the EMG analysis quantified action intensity rather than fatigue-related spectral change. These works show that haptic feedback can improve interaction quality while simultaneously creating repeated motor demands that are not captured by conventional usability outcomes.

Fatigue-related outcomes in VR are analytically distinct across constructs. Luo et al. examined cognitive fatigue caused by visual–proprioceptive, visual–vestibular, and visual–experiential conflicts during VR remapping using subjective ratings, task performance, and EEG [21]. Visual–vestibular conflict produced the earliest and most severe cognitive-fatigue response, whereas a salient visual–experiential manipulation sometimes delayed fatigue by increasing novelty and engagement. This work is relevant to the design of immersive tasks, but its outcome is sustained cognitive depletion rather than a reduction in the force-generating capacity of a local muscle group. Similarly, bandwidth-related mental fatigue in multimedia communication [34] and clinical fatigue syndromes [35] are not direct physiological equivalents of localized upper-limb fatigue.

A complementary research direction uses haptic stimulation as an intervention rather than treating haptics only as a source of workload. Lin et al. developed a skin-integrated actuator combining mechanical force and electrical stimulation and evaluated recovery with sEMG frequency measures [26]. Galofaro et al. used neuromuscular electrical stimulation to render haptic load in virtual reality and compared kinematics, metabolic effort, and perceived fatigue with physical and visual-only conditions [36]. These studies show that haptic feedback can alter physical demand as well as interaction quality.

### 2.3. Health 5.0 Medical Robotics and Fatigue-Aware Haptic Interaction

Health 5.0 medical robotics places fatigue monitoring within a safety- and care-oriented human–robot loop. In robot-assisted minimally invasive surgery, the surgeon interacts continuously with a master console rather than directly with the instruments. This arrangement can improve posture and reduce fatigue in some muscle groups, but it does not eliminate ergonomic exposure. Armijo et al. recorded upper-trapezius, anterior-deltoid, flexor-carpi-radialis, and extensor-digitorum sEMG during laparoscopic and robotic procedures and found technique-dependent differences in activation and MDF rather than uniformly absent fatigue [7]. Pérez-Salazar et al. combined EMG, kinematics, electrodermal activity, and electrocardiography during conventional and robotic-assisted surgical tasks; robotic assistance improved several ergonomic outcomes, while muscle-specific loading and stress remained measurable [8]. Wearable EMG has also revealed fatigue accumulation across phases of a prolonged surgical procedure and has been proposed to guide ergonomic adjustment and micro-breaks [37]. These findings support continuous clinician-state monitoring even when robotics improves the average ergonomics of surgery.

Patient-facing rehabilitation robots create a related but physiologically different problem. The robot may assist movement, resist movement, or render a target trajectory through haptic forces. During assist-as-needed upper-limb training with a HapticMaster robot, Poyil et al. observed fatigue-related decreases in median frequency and changes in the relation between EMG and interaction force, showing that fatigue can alter both the biological signal and the control information used by the robot [4]. Covaciu et al. subsequently described a robot-assisted rehabilitation protocol that tracks MDF and RMS and initiates a fatigue check when EMG parameters deteriorate [5]. These studies motivate adaptive medical robots that distinguish productive therapeutic effort from excessive loading and modify assistance, resistance, repetitions, or rest under clinician-defined safety constraints.

The current study investigates interaction primitive commonly observed across the tested scenarios: sustained, repetitive manipulation of the hand–arm against computer-controlled forces, along with the acquisition of multimuscle sEMG, kinematics, and force. This experiment provides a methodological framework for evaluating an arm-level spectral summary related to teleoperation by a clinician and rehabilitation.

Other relevant works in MDPI publications address the following topics: wearable sensing, virtual environments, medical robotics, occupational fatigue, and Health 5.0 monitoring. Caporaso et al. integrated five sEMG sensors and an accelerometer into a virtual reality system for ergonomic assessment of human–robot cooperation at the workplace [2]. Brambilla et al. presented the state-of-the-art of upper limb fatigue, strain, and effort assessment, ranging from laboratory protocol-based testing to field testing in industry and found that electromyography (EMG) is the most popular instrumental approach in laboratory studies [38]. Moyen-Sylvestre et al. showed that spectral changes associated with fatigue occur in the acceleration and angular velocity of low-load repetitive arm movements [39]. Merbah et al. used wearable EMG sensors to quantify the muscular fatigue during surgical operations and demonstrated the importance of specific visualization of muscles’ fatigue in professional tasks [37]. Kuber et al. used a combination of EMG and inertial features to classify the perceived fatigue in exoskeleton-assisted industrial tasks [40]. Daniel et al. discussed problems in the methodology of dynamic sEMG fatigue assessment and highlighted electrode displacement, changes in contraction conditions, and the need for a rigorously controlled analysis pipeline [41]. In the context of Health 5.0, Scano et al. described the growing popularity of wearable physiological sensors, including sEMG, in health and rehabilitation applications [15], while Oliveira et al. emphasized the need for continuous and personalized monitoring in Health 5.0 [14]. The Table 1 provides an overview of the most relevant works in medical robotics, rehabilitation, surgery, virtual interaction, and occupational fatigue sensing, along with the current gaps.

Together, these studies establish the relevance of wearable sensing to fatigue assessment in surgery, rehabilitation, industrial work, and human-centered system design, but they do not resolve how to summarize fatigue-related spectral change across several upper-limb muscles during repetitive force–feedback interaction. Recent comparative work also shows that no single sEMG fatigue index should be assumed to dominate across dynamic tasks because feature behavior depends on movement, preprocessing, and the chosen fatigue definition [29,41]. The present study therefore makes a task-specific comparison of MNF- and MDF-derived arm indices and evaluates not only their association with an archived fatigue-analysis score, but also participant influence, uncalibrated scale correspondence, and leave-one-participant-out calibration.

## 3. Materials and Methods

### 3.1. Study Design and Participants

This laboratory study used a single-session observational protocol to characterize upper-limb neuromuscular fatigue during a prolonged haptic-writing task. The experiment was conducted at the University of Ottawa after institutional ethics approval had been obtained. Twenty adults voluntarily participated (12 men and 8 women; age range, 25–59 years). Before enrollment, each participant received an explanation of the study procedures and provided written informed consent. Before data collection, each participant completed two familiarization trials. The archived demographic variable distinguished men and women. No separate gender-identity, anthropometric-strength, or hormonal-status variables were available; therefore, any subgroup description is treated as exploratory and sex-stratified rather than as a definitive gender analysis.

The study was designed as an exploratory physiological characterization rather than as a powered superiority or clinical-validation trial. Consistent with precision-oriented guidance for pilot studies, the fixed N=20 cohort is therefore justified by the proof-of-concept objective and evaluated through effect estimates and confidence intervals rather than retrospective power calculations [42]. For a correlation estimated from N=20, the Fisher-*z* standard error is $1/\sqrt{N-3}=0.243$; the observed 95% intervals (0.885–0.982 for MDF and 0.521–0.910 for MNF) make the resulting precision explicit. To assess whether the MDF-MNF ordering was driven by any single participant, we additionally performed a leave-one-participant-out influence analysis in which both correlations and Δr were recomputed 20 times. The cohort is thus used to characterize signal behavior, estimate effect magnitude, and inform a prospectively powered replication, not to claim population-level validation.

### 3.2. Experimental Apparatus and Haptic Environment

**Figure 2:**
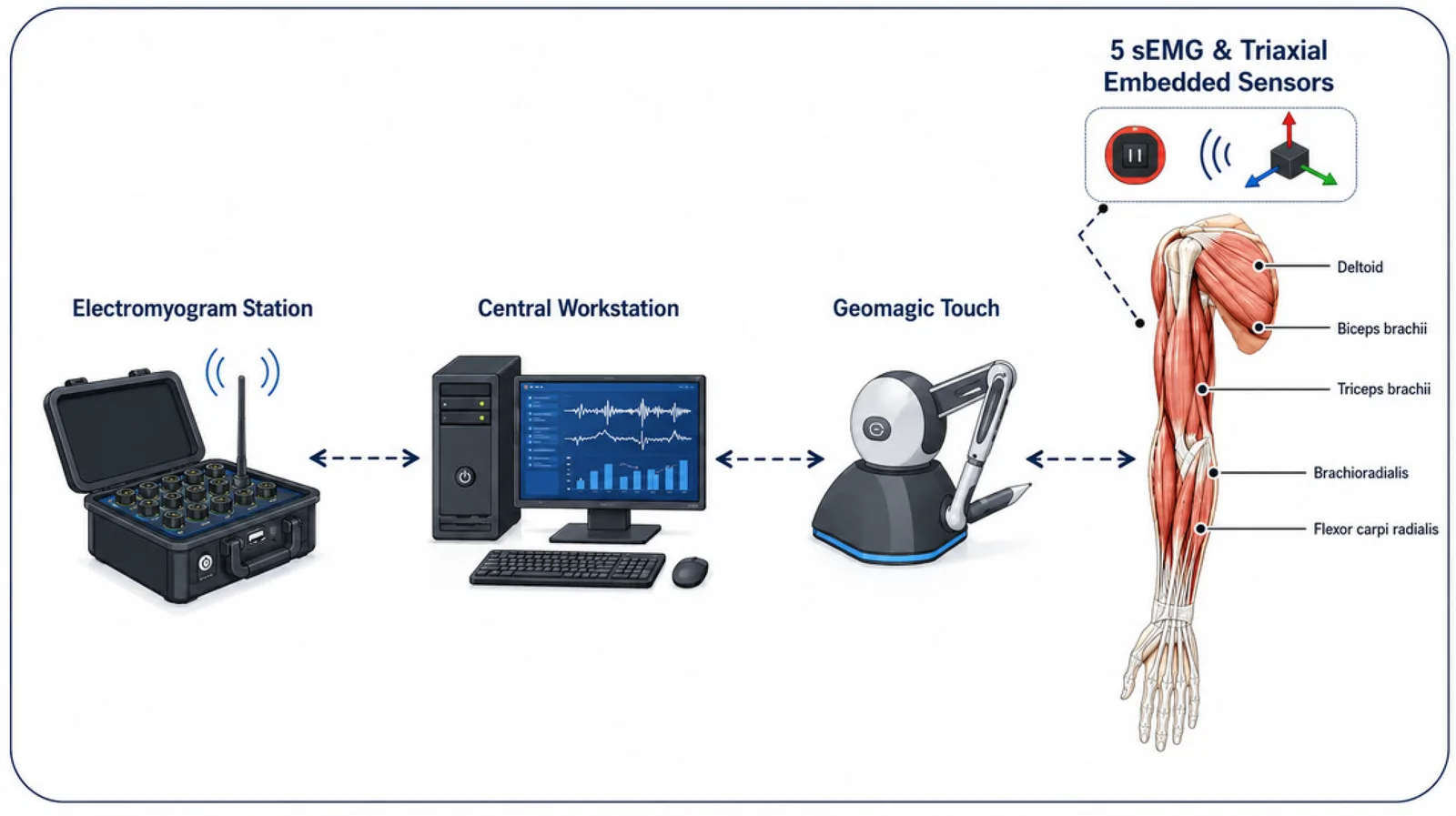
Architecture of the experimental haptic–sEMG platform. Five wireless surface-EMG sensors with embedded triaxial accelerometers were positioned over the deltoid, biceps brachii, triceps brachii, brachioradialis, and flexor carpi radialis. The electromyogram station communicated with the central workstation and the Geomagic Touch haptic interface.

**Figure 3:**
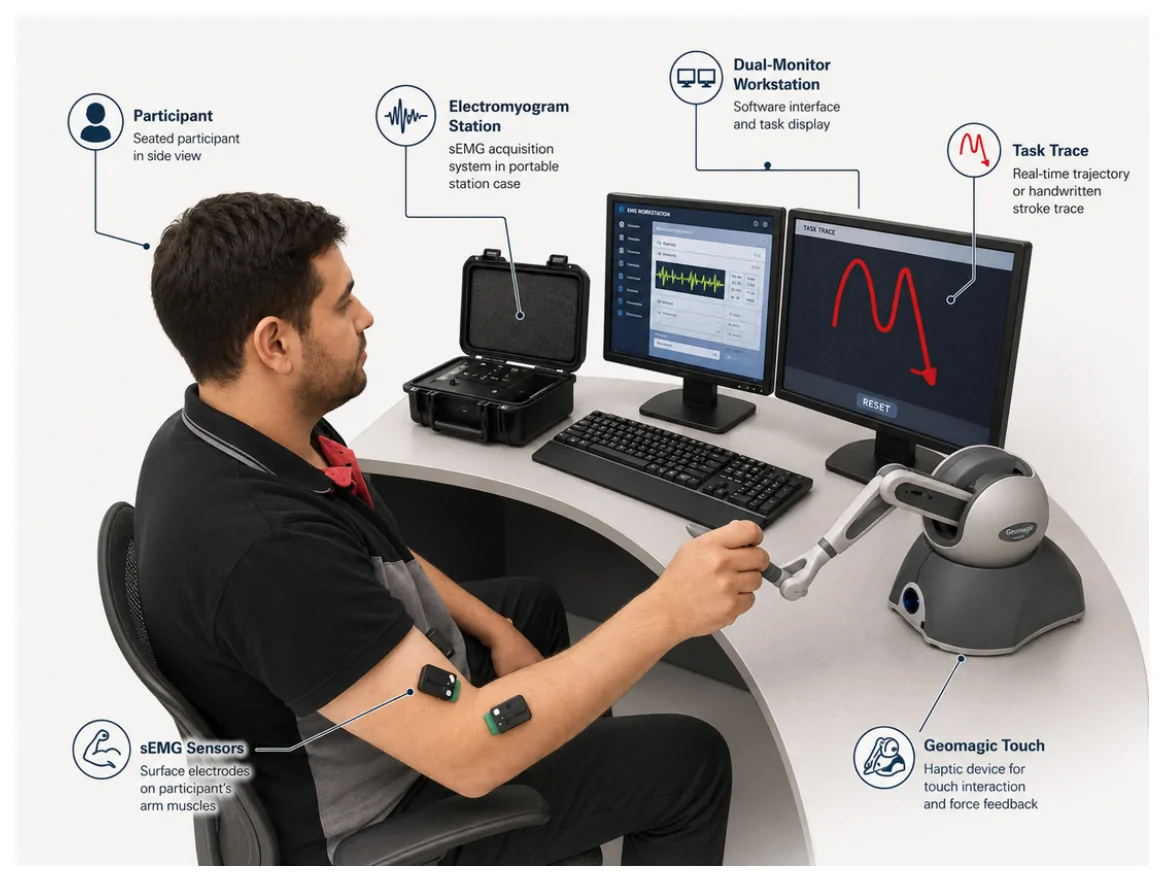
Representative experimental configuration. The participant manipulated the Geomagic Touch stylus while viewing the target trace on a dual-monitor workstation. Wireless sEMG sensors recorded upper-limb activity.

The experimental platform combined a Geomagic Touch force–feedback device, a wireless Delsys sEMG system with embedded triaxial accelerometers, and a central workstation (Figure 2). The haptic device provided six-degree-of-freedom position sensing and three-degree-of-freedom force feedback [43]. The writing environment was implemented in Unity (https://unity.com/cn) using C# and a haptic plug-in [44]. It displayed a target Arabic character, recorded the stylus trajectory, and rendered contact forces. The end-effector interaction reproduced generic features relevant to medical-robot master consoles and rehabilitation end effectors, including repeated hand–arm control, computer-rendered resistance, and synchronized force and movement acquisition. These shared features provide a controlled basis for translational hypothesis generation. Figure 3 shows the participant configuration and task display.

### 3.3. Muscles, Sensor Placement, and Experimental Protocol

Wireless sensors were positioned over the flexor carpi radialis, brachioradialis, triceps brachii, biceps brachii, and deltoid. These muscles were selected to represent wrist control, elbow flexion–extension, and shoulder stabilization during stylus manipulation. The skin was cleaned with rubbing alcohol and the sensors were secured over the central muscle region [45]. This approach is consistent with the general SENIAM principles of placing bipolar surface electrodes over the muscle belly, away from tendinous regions and adjacent muscle borders, to improve reproducibility and reduce cross-talk [46]. Study-specific locations followed the experimental protocol and the fixed geometry of the Delsys sensors.

After familiarization, participants repeatedly reproduced a prescribed Arabic character and a signature-like movement for 400 s. Three-dimensional stylus position, interaction force, sEMG, and triaxial acceleration were recorded throughout the task. The protocol preserved the self-selected timing, force modulation, and movement strategies that characterize continuous VR interaction. The resulting indices quantify fatigue-related spectral change during natural task execution. Position, force, and acceleration were retained as contextual measurements when interpreting the sEMG trajectories. For Health 5.0 interpretation, the exposure represents a repeated haptic-control task relevant to medical training, telemanipulation, and rehabilitation scenarios. Immediately after the task, participants completed a six-item questionnaire covering fatigue, arm-fatigue level, realism, usefulness, overall experience, and frustration/boredom.

### 3.4. Notation and Recorded Signals

Participants are indexed by p∈{1,…,N}, muscles by i∈{1,…,K}, and discrete samples by *n*. In this study, N=20 and K=5. The raw sEMG signal from participant *p* and muscle *i* is denoted by $s_{p,i}\left[n\right]$, and the sampling frequency is $f_{s}=1000$ Hz. The filtered sEMG is $x_{p,i}\left[n\right]$. Triaxial force samples from the haptic interface are $F_{x,p}\left[n\right]$, $F_{y,p}\left[n\right]$, and $F_{z,p}\left[n\right]$, and the resultant force is

(1) $$F_{\mathrm{res},p}\left[n\right]=\sqrt{F_{x,p}{\left[n\right]}^{2}+F_{y,p}{\left[n\right]}^{2}+F_{z,p}{\left[n\right]}^{2}}.$$

Here, $F_{\mathrm{res},p}\left[n\right]$ is expressed in newtons and represents the instantaneous magnitude of the three-dimensional interaction force. Position and acceleration were analyzed descriptively to contextualize changes in task mechanics; neither was treated as a direct measure of fatigue.

### 3.5. sEMG Preprocessing and Windowing

For notation, the archived preprocessing pipeline is represented by

(2) $$x_{p,i}\left[n\right]=B\;\left\{s_{p,i}\left[n\right]-{\bar{s}}_{p,i}\right\},$$

where ${\bar{s}}_{p,i}$ is the recording mean and B{·} denotes the Bessel filtering and power-line-removal operation used in the original analysis. The historical record confirms the filter family and power-line-removal step. The numerical settings are preserved as historical provenance rather than retrospectively reassigned; Table 2 therefore pairs the retained historical information with a fully specified prospective replication and sensitivity plan. The set $Q_{p,i}$ denotes the windows retained by the original pipeline; excluded windows were omitted rather than replaced.

The analysis used 0.125 s windows with approximately 50% overlap. At $f_{s}=1000$ Hz, the window length was therefore

(3) $$L=\left\lfloor 0.125f_{s}\right\rfloor =125\;\mathrm{samples},\;H=\left\lfloor 0.0625f_{s}\right\rfloor =62\;\mathrm{samples},$$

where *L* is the number of samples per window and *H* is the hop size between successive windows. This gives 63 shared samples, or 50.4% overlap. For window index q=0,…,Q−1 and within-window sample ℓ=0,…,L−1,

(4) $$x_{p,i,q}\left[\ell \right]=x_{p,i}\left[qH+\ell \right],\;Q=1+\left\lfloor \frac{N_{s}-L}{H}\right\rfloor ,$$

where $N_{s}$ is the number of samples in the recording and *Q* is the number of complete analysis windows. A Hann taper w[ℓ] was applied before spectral estimation.

### 3.6. Power-Spectral Density Estimation

The discrete Fourier transform of window *q* was

(5) $$X_{p,i,q}\left[j\right]=\sum_{\ell =0}^{L-1}w\left[\ell \right]x_{p,i,q}\left[\ell \right]\exp\;\left(-i\frac{2\pi j\ell }{L}\right),$$

where *j* is the frequency-bin index and $i=\sqrt{-1}$. The corresponding one-sided periodogram was

(6) $$P_{p,i,q}\left[j\right]=\frac{2}{f_{s}U}{\left|X_{p,i,q}\left[j\right]\right|}^{2},\;U=\sum_{\ell =0}^{L-1}w{\left[\ell \right]}^{2},$$

with the usual exception that the direct-current and Nyquist bins are not doubled. The frequency represented by bin *j* was

(7) $$f_{j}=\frac{jf_{s}}{L},\;\Delta f=\frac{f_{s}}{L}=8\;\mathrm{Hz},$$

where Δf is the frequency resolution. Spectral features were computed over the retained-bin set

(8) $$J=\left\{j:f_{L}\leq f_{j}\leq f_{U}\right\},$$

where $f_{L}$ and $f_{U}$ are the lower and upper analysis-band limits. A provenance audit of the earlier peer-reviewed implementation confirmed Bessel filtering, 1 kHz acquisition, and the 0.125 s/0.0625 s window/overlap specification [31]. That report did not state the Bessel order, numerical cutoffs, power-line-removal implementation, or numerical $\left[f_{L},f_{U}\right]$ limits. Rather than retroactively assigning those missing values to the archived participant indices, Table 2 separates settings that are historically documented from unresolved parameters and from a fully specified prospective replication pipeline. This provenance-based treatment makes the reproducibility boundary explicit and prevents conventional defaults from being misrepresented as historical facts.

For avoidance of ambiguity, the Butterworth/Bessel orders and cutoffs, the 20–400/20–450 Hz numerical bands, the prospective QC thresholds, and the 250/500 ms window options in Table 2 are recommendations for future reprocessing only; they were not used to compute the historical participant-level MNF/MDF indices or any reported participant-level results.

### 3.7. Mean and Median Frequency

For each valid analysis window, mean frequency was calculated as the power-weighted spectral centroid,

(9) $${\mathrm{MNF}}_{p,i,q}=\frac{\sum_{j\in J}f_{j}P_{p,i,q}\left[j\right]}{\sum_{j\in J}P_{p,i,q}\left[j\right]}.$$

Here, ${\mathrm{MNF}}_{p,i,q}$ is measured in hertz and summarizes the location of the complete retained power spectrum.

Median frequency was defined by the half-power condition. Let

(10) $$C_{p,i,q}\left[k\right]=\sum_{\begin{array}{c} j\in J\\ j\leq k \end{array}}P_{p,i,q}\left[j\right],\;P_{p,i,q}^{\mathrm{tot}}=\sum_{j\in J}P_{p,i,q}\left[j\right],$$

and let $k^{*}$ be the smallest retained bin satisfying

(11) $$C_{p,i,q}\left[k^{*}\right]\geq \frac{1}{2}P_{p,i,q}^{\mathrm{tot}}.$$

To reduce quantization caused by the 8 Hz bin spacing, MDF was estimated by linear interpolation within the crossing bin. Let $k_{0}=\min J$ denote the first retained bin, let

(12) $$C_{p,i,q}^{-}\left[k\right]=\sum_{\begin{array}{c} j\in J\\ j<k \end{array}}P_{p,i,q}\left[j\right],\;C_{p,i,q}^{-}\left[k_{0}\right]=0,$$

and define the retained lower and upper edges of bin *k* as

(13) $$\ell_{k}=\max\;\left(f_{L},f_{k}-\frac{\Delta f}{2}\right),\;u_{k}=\min\;\left(f_{U},f_{k}+\frac{\Delta f}{2}\right).$$

The interpolated estimate is

(14) $${\mathrm{MDF}}_{p,i,q}=\ell_{k^{*}}+\left(u_{k^{*}}-\ell_{k^{*}}\right)\frac{\frac{1}{2}P_{p,i,q}^{\mathrm{tot}}-C_{p,i,q}^{-}\left[k^{*}\right]}{P_{p,i,q}\left[k^{*}\right]}.$$

The crossing condition guarantees that the interpolation fraction lies in [0,1] for every nonzero-power accepted window; consequently, the estimate remains inside the retained crossing bin. When $k^{*}=k_{0}$, Equation (14) uses $C^{-}\left[k_{0}\right]=0$ and the retained lower band edge $\ell_{k_{0}}=f_{L}$, eliminating the undefined preceding-bin reference. Relative to another within-bin edge convention, the maximum change in an affected window is bounded by the width of the first retained bin (at most 8 Hz here). Both MNF and MDF were interpreted as fatigue-sensitive spectral features rather than direct measurements of force-generating capacity [22,23,24].

### 3.8. Spectral-Trajectory Model and Muscle-Level Fatigue Index

Let $z_{p,i,q}$ denote either ${\mathrm{MNF}}_{p,i,q}$ or ${\mathrm{MDF}}_{p,i,q}$. To make polynomial coefficients independent of the 400 s duration, window-center time $t_{q}$ was normalized to

(15) $$\tau_{q}=\frac{t_{q}-t_{0}}{t_{Q-1}-t_{0}},\;0\leq \tau_{q}\leq 1.$$

A second-order model was fitted separately for every participant, muscle, and spectral feature,

(16) $${\hat{z}}_{p,i}\left(\tau \right)=a_{p,i,z}\tau^{2}+b_{p,i,z}\tau +c_{p,i,z},$$

where $a_{p,i,z}$ is the curvature coefficient, $b_{p,i,z}$ is the linear coefficient, and $c_{p,i,z}$ is the fitted value at the beginning of the task. The coefficients were estimated by least squares over quality-controlled windows,

(17) $$\left({\hat{a}}_{p,i,z},{\hat{b}}_{p,i,z},{\hat{c}}_{p,i,z}\right)=\underset{a,b,c}{\arg\;\min}\sum_{q\in Q_{p,i}}{\left[z_{p,i,q}-\left(a\tau_{q}^{2}+b\tau_{q}+c\right)\right]}^{2},$$

where $Q_{p,i}$ is the set of accepted windows for participant *p* and muscle *i*.

The second derivative, $d^{2}\hat{z}/d\tau^{2}=2a_{p,i,z}$, represents trajectory curvature rather than percentage decline. The muscle-level fatigue index was therefore defined as the signed fitted start-to-end decrease,

(18) $$\begin{array}{cc} {\mathrm{FI}}_{p,z}^{\left(i\right)}\; & =100\;\frac{{\hat{z}}_{p,i}\left(0\right)-{\hat{z}}_{p,i}\left(1\right)}{{\hat{z}}_{p,i}\left(0\right)}\\ \; & =-100\;\frac{a_{p,i,z}+b_{p,i,z}}{c_{p,i,z}},\;z\in \left\{\mathrm{MNF},\mathrm{MDF}\right\}. \end{array}$$

A positive value indicates a downward spectral shift, zero indicates no fitted change, and a negative value indicates an increase. The absolute value is not taken because doing so would incorrectly classify upward shifts as fatigue. Curvature $2a_{p,i,z}$ is a separate trajectory descriptor and is not part of the percentage fatigue index.

Although Equation (18) is evaluated at τ=0 and τ=1, it is not a two-sample endpoint calculation: $a_{p,i,z}$, $b_{p,i,z}$, and $c_{p,i,z}$ are estimated from every accepted window in $Q_{p,i}$. The quadratic was used as the lowest-order nonlinear smoother that permits one change in slope, allowing an early decline followed by a plateau or a late acceleration while avoiding the additional oscillations available to higher-order polynomials. The resulting index quantifies whether the fitted spectrum is lower at the end of the exposure than at the beginning. Trajectory-shape descriptors were retained separately so that net decline and within-task curve shape remain distinct.

Trajectory shape is separable from net decline. The fitted derivative and any interior turning point are

(19) $$g_{p,i,z}\left(\tau \right)=2a_{p,i,z}\tau +b_{p,i,z},\;\tau_{p,i,z}^{*}=-\frac{b_{p,i,z}}{2a_{p,i,z}}\;\left(a_{p,i,z}\neq 0\right).$$

The fitted curve is monotonic non-increasing over [0,1] when $b_{p,i,z}\leq 0$ and $2a_{p,i,z}+b_{p,i,z}\leq 0$; a turning point satisfying $0<\tau_{p,i,z}^{*}<1$ indicates non-monotonic fitted behavior. Model adequacy is summarized by

(20) $$R_{p,i,z}^{2}=1-\frac{\sum_{q\in Q_{p,i}}{\left[z_{p,i,q}-{\hat{z}}_{p,i}\left(\tau_{q}\right)\right]}^{2}}{\sum_{q\in Q_{p,i}}{\left[z_{p,i,q}-{\bar{z}}_{p,i}\right]}^{2}},$$

where ${\bar{z}}_{p,i}$ is the mean spectral value over accepted windows. The participant-level analysis focused on the final muscle and arm indices; the reported FI is therefore interpreted as net fitted spectral decline.

### 3.9. Arm-Level Fatigue Index

The participant-level arm index was the unweighted average of the five muscle-specific indices,

(21) $${\mathrm{FI}}_{p,z}^{\mathrm{arm}}=\frac{1}{K}\sum_{i=1}^{K}{\mathrm{FI}}_{p,z}^{\left(i\right)},\;K=5.$$

**Figure 4:**
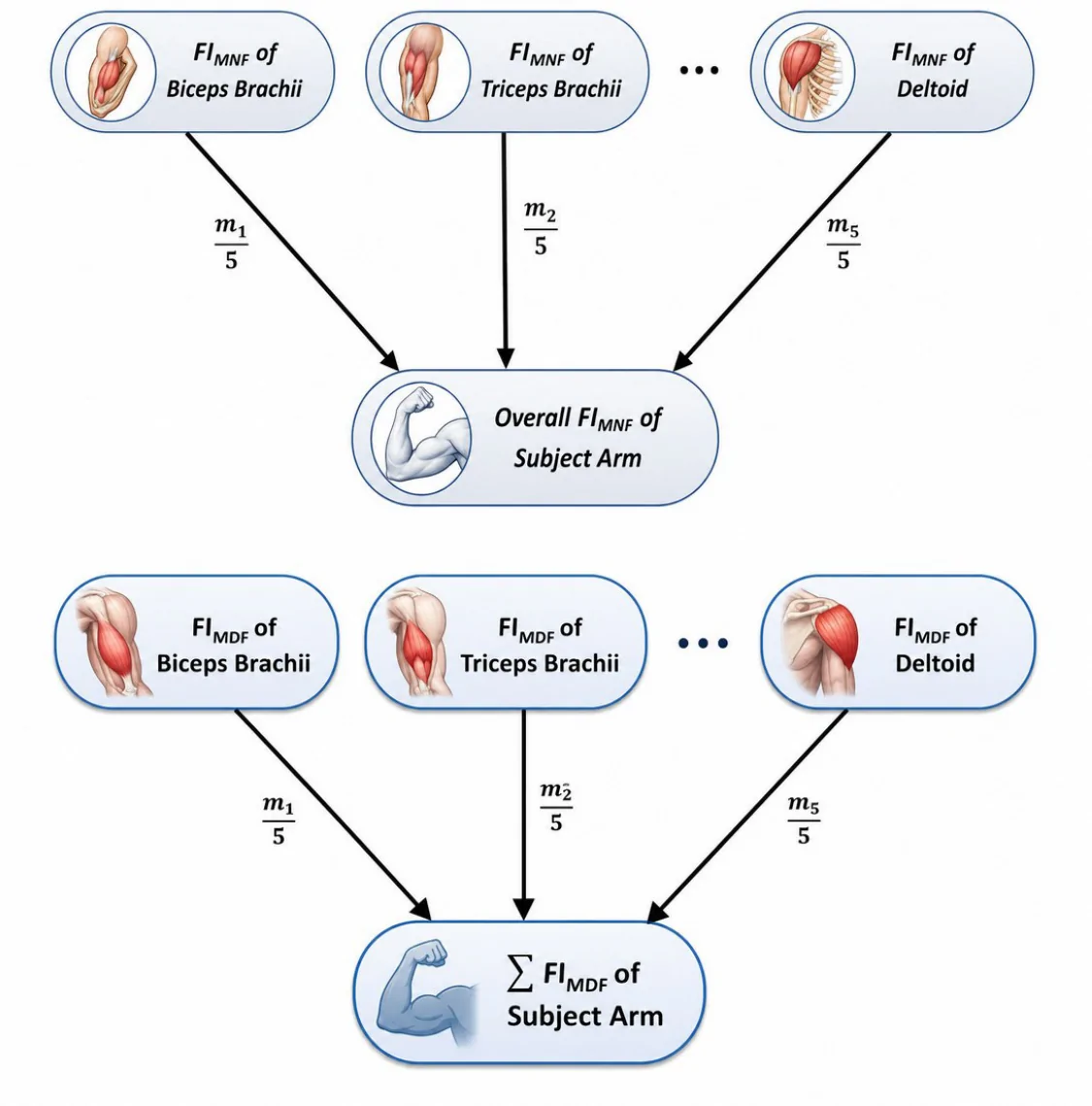
Construction of the participant-level arm-fatigue indices from the five muscle-specific indices. The upper diagram shows MNF-derived aggregation and the lower diagram shows MDF-derived aggregation. Each valid muscle contributes 1/5 of the arm-level index; $m_{1},\ldots ,m_{5}$ denote the five monitored muscles.

Equal weighting was used because no validated task-specific physiological weighting scheme was available and because it avoids assigning outcome-dependent importance to individual muscles. Valid indices were available for all five muscles in every participant. Figure 4 summarizes the equal-weight multimuscle aggregation used to construct the participant-level MNF- and MDF-derived arm indices. Table 3 summarizes the principal notation used in the signal-processing and fatigue-index equations. Equal weighting is therefore a transparent, outcome-independent aggregation rule, not evidence that all five muscles contribute equally to stylus biomechanics. A direct leave-one-muscle-out robustness analysis requires the participant-by-muscle FI matrix rather than the retained arm-level averages. Table 4 therefore prespecifies five leave-one-muscle-out recomputations and an activation-weighted sensitivity analysis using independently normalized RMS activation when such normalization data are available; no weights may be optimized against $Y_{p}$.

### 3.10. Computational Algorithms

Algorithm 1 summarizes the participant-by-muscle feature-extraction pipeline. Retained windows were Hann tapered and converted to one-sided periodograms. MNF and interpolated MDF were calculated over the retained spectral bins, and a quadratic trajectory was fitted to each frequency series over normalized task time. Equation (18) converts each fitted trajectory into a signed muscle-level net-decline index.

Algorithm 2 defines the arm-level aggregation and archived-score comparison stage. Equation (21) averaged the five muscle indices to obtain participant-level MNF and MDF arm indices. Index construction was completed independently of $Y_{p}$. Participant-level association, exploratory uncalibrated scale-correspondence, bootstrap, randomization, participant-influence, and internal-calibration analyses were subsequently applied to the fixed arm indices.
**Algorithm 1** MNF/MDF extraction and muscle-level fatigue estimation**Require:** $s_{p,i}\left[n\right]$, $f_{s}$, *L*, *H*, and $\left[f_{L},f_{U}\right]$ **Ensure:** ${\mathrm{FI}}_{p,\mathrm{MNF}}^{\left(i\right)}$ and ${\mathrm{FI}}_{p,\mathrm{MDF}}^{\left(i\right)}$
  1:Remove the recording mean and apply the documented filter operator B  2:Segment the signal into length-*L* windows separated by hop *H*  3:**for** each window *q* **do**  4:    Apply the Hann taper and window-retention criteria  5:    **if** window *q* is retained **then**  6:        Compute $X_{p,i,q}\left[j\right]$ and $P_{p,i,q}\left[j\right]$  7:        Compute ${\mathrm{MNF}}_{p,i,q}$ using Equation (9)  8:        Compute ${\mathrm{MDF}}_{p,i,q}$ using Equations (11)–(14)  9:    **end if**10:**end for**11:Normalize accepted window times to τ∈[0,1]12:**for** z∈{MNF,MDF} **do**13:    Fit ${\hat{z}}_{p,i}\left(\tau \right)=a\tau^{2}+b\tau +c$14:    Compute ${\mathrm{FI}}_{p,z}^{\left(i\right)}=-100\left(a+b\right)/c$15:**end for**


**Algorithm 2** Arm-level aggregation and archived-score comparison**Require:** Five valid muscle indices and archived fatigue-score variable $Y_{p}$
**Ensure:** Arm indices, association, exploratory scale-correspondence, and calibration statistics
  1:**for** each participant *p* **do**  2:    **for** z∈{MNF,MDF} **do**  3:        ${\mathrm{FI}}_{p,z}^{\mathrm{arm}}\leftarrow K^{-1}\sum_{i=1}^{K}{\mathrm{FI}}_{p,z}^{\left(i\right)}$  4:        $e_{p,z}\leftarrow {\mathrm{FI}}_{p,z}^{\mathrm{arm}}-Y_{p}$  5:    **end for**  6:**end for**  7:Compute Pearson and Spearman associations between each arm index and $Y_{p}$  8:Compare the dependent MDF–$Y_{p}$ and MNF–$Y_{p}$ correlations  9:Apply participant bootstrap and paired label-swap randomization sensitivity analyses10:Compute MAE, RMSE, and Bland–Altman-form difference diagnostics only as exploratory uncalibrated scale correspondence11:Evaluate difference normality and proportional bias12:Compare paired absolute errors and apply the prespecified multiplicity rule


### 3.11. Analysis-Parameter Handling and Outcome Independence

The 0.125 s window, 0.0625 s overlap, and second-order trajectory model were documented in the feature-extraction stage before the archived-score comparison. The quadratic form represented the expected nonlinear spectral-decay pattern rather than an explicit optimization against $Y_{p}$. In the present participant-level reanalysis, the window specification, spectral-feature definitions, aggregation rule, and model form were held fixed and applied identically to MNF and MDF; no parameter retuning was performed using $Y_{p}$.

The statistical reanalysis used fixed participant-level MNF and MDF index values; therefore, $Y_{p}$ did not influence raw-signal processing in this reanalysis. The comparison therefore used the fixed participant-level indices generated by the processing pipeline.

The historical data set does not include the participant-by-muscle/window matrix, fitted channel-level start/end frequencies, complete QC logs, or participant-linked mechanical summaries; therefore, the requested muscle-level, leave-one-muscle-out, longer-window/model, channel-plausibility, and mechanically adjusted sensitivity analyses could not be performed without introducing unsupported data.

### 3.12. Questionnaire Measure and Archived Fatigue-Score Provenance

Immediately after the 400 s haptic-writing task, participants completed the six-item questionnaire shown in Table 5. The arm-fatigue item was Q2: “Please indicate your current arm fatigue level?” Responses were recorded on a seven-category numerical rating scale rather than a visual analogue scale. The endpoints were 1 (“not at all”) and 7 (“completely”), with higher values indicating greater arm fatigue; the intermediate categories were not verbally labeled.

Two distinct fatigue data products are available and are now analyzed separately. The raw Q2 questionnaire distribution contains scores 5, 6, and 7 with counts 2, 9, and 9 (mean 6.35/7). A direct linear rescaling by 100(r−1)/6 would therefore have an aggregate mean of 89.17/100. The archived participant-level analysis variable $Y_{p}$, in contrast, ranges from 25 to 90 with a mean of 56.95. These distributions are not numerically equivalent, so the revised analysis does not treat $Y_{p}$ as a direct linear recoding of the raw Q2 categories. Raw Q2 is used for questionnaire descriptives, whereas $Y_{p}$ is retained as the legacy participant-level analysis criterion for the historical objective–score comparison. Accordingly, the manuscript no longer labels $Y_{p}$ as a transformed Q2 score. All $Y_{p}$ correlations, calibration results, numerical-error summaries, and difference plots are explicitly identified as exploratory analyses of the archived analysis variable; the raw Q2 distribution remains a separate questionnaire outcome.

To make this separation testable rather than merely verbal, Table 6 compares the raw Q2 category counts with the category distribution obtained by projecting each archived $Y_{p}$ value to the nearest admissible seven-category percentage. The projected distribution (scores 2–6: 1, 3, 7, 6, and 3 participants) differs markedly from the recorded raw Q2 distribution (scores 5–7: 2, 9, and 9 participants). Thus, nearest-category projection is retained only as a numerical-resolution stress test; it is not used as reconstructed questionnaire data.

For reference, a direct linear rescaling of a documented raw Q2 category $r_{p,2}\in \left\{1,\ldots ,7\right\}$ would be

(22) $$C_{p}=100\frac{r_{p,2}-1}{6},$$

which yields

(23) C={0,16.67,33.33,50,66.67,83.33,100}.

Equations (22) and (23) define only the mathematical rescaling of a known raw category; they are not asserted to be the historical transformation that generated $Y_{p}$.

The descriptive questionnaire distributions were calculated from the archived category counts. For item *h* and response category k∈{1,…,7},

(24) $$P_{h,k}=100\frac{n_{h,k}}{N},\;\sum_{k=1}^{7}P_{h,k}=100\%,$$

where $n_{h,k}$ is the number of participants selecting category *k* and N=20.

A seven-level discretization stress test was retained only to determine whether the MDF-MNF point-estimate ordering was highly dependent on the fine numerical resolution of $Y_{p}$. Each $Y_{p}$ value was projected to the nearest value in C,

(25) $${\tilde{C}}_{p}=\underset{c\in C}{\arg\;\min}\;\left|Y_{p}-c\right|.$$

This operation is not a reconstruction of the participants’ raw Q2 responses and is not used to reconcile the provenance discrepancy.

### 3.13. Statistical Analysis, Influence, Calibration, and Scale Correspondence

For spectral feature *z*, the participant-level error was

(26) $$e_{p,z}={\mathrm{FI}}_{p,z}^{\mathrm{arm}}-Y_{p}.$$

Mean absolute error and root-mean-square error were

(27) $${\mathrm{MAE}}_{z}=\frac{1}{N}\sum_{p=1}^{N}\left|e_{p,z}\right|,\;{\mathrm{RMSE}}_{z}=\sqrt{\frac{1}{N}\sum_{p=1}^{N}e_{p,z}^{2}}.$$

Association was summarized with Pearson correlation and Fisher-transformed 95% confidence intervals; Spearman correlation was used as a rank-based sensitivity analysis. Because the MDF–$Y_{p}$ and MNF–$Y_{p}$ correlations were calculated from the same participants and shared $Y_{p}$, their difference was tested directly with Steiger’s *z* test for two dependent, overlapping correlations, incorporating the observed MNF–MDF intercorrelation [47]. The effect was reported as $\Delta r=r_{\mathrm{MDF},Y}-r_{\mathrm{MNF},Y}$ with a 95% confidence interval calculated using Zou’s method [48]. Participant-level nonparametric bootstrap resampling (100,000 replicates; fixed random seed) provided an additional interval for Δr. A paired label-swap randomization analysis enumerated all $2^{20}$ within-participant exchanges of the MNF and MDF labels and compared the observed absolute Δr with the resulting randomization distribution. This test evaluates the stronger exchangeability null that the two index labels are interchangeable within participant; it was therefore interpreted as a conservative sensitivity analysis rather than as a replacement for the Steiger test.

Exploratory uncalibrated scale correspondence was evaluated with a Bland–Altman-form difference diagnostic [49]. The arm FI is a percentage spectral-decline index and $Y_{p}$ is an archived 0–100 analysis score; therefore, a raw one-to-one comparison is informative about offset and dispersion but is not assumed a priori to be a calibrated measurement substitution. For each participant,

(28) $$d_{p,z}=e_{p,z},\;m_{p,z}=\frac{{\mathrm{FI}}_{p,z}^{\mathrm{arm}}+Y_{p}}{2},$$

where $d_{p,z}$ is the paired difference and $m_{p,z}$ is the paired mean. Bias and conventional 95% limits of agreement were

(29) $${\bar{d}}_{z}=\frac{1}{N}\sum_{p=1}^{N}d_{p,z},\;{\mathrm{LoA}}_{z}={\bar{d}}_{z}\pm 1.96s_{d,z},$$

where $s_{d,z}$ is the sample standard deviation of the differences. The conventional limits are reported only as descriptive difference bounds on the archived scale; their usual method-agreement interpretation is not invoked because FI and $Y_{p}$ are not established as commensurate measurement methods. The calculation assumes that the differences are approximately normally distributed and do not vary systematically over the numerical range. These assumptions were examined using a Shapiro–Wilk diagnostic for the differences and a regression of $d_{p,z}$ on $m_{p,z}$ for proportional bias,

(30) $$d_{p,z}=\gamma_{0,z}+\gamma_{1,z}m_{p,z}+u_{p,z}.$$

Because normality can be fragile with N=20 and both scales are bounded, conventional limits were supplemented by the empirical 2.5th and 97.5th percentiles of the observed differences and by the seven-level discretization stress test. Participant-level bootstrap resampling provided confidence intervals for Δr, error metrics, bias, and conventional limits of agreement.

The inferential hierarchy was defined before the present reanalysis. The single planned inferential comparison was the difference between the dependent MDF–$Y_{p}$ and MNF–$Y_{p}$ Pearson correlations. The paired difference in absolute error was the only formal secondary comparison. The six questionnaire items were summarized descriptively, while Spearman correlation, MAE, RMSE, MAPE, bias, limits of agreement, seven-level discretization, normality diagnostics, proportional-bias diagnostics, randomization analyses, participant-influence analysis, and internal calibration were treated as complementary effect summaries or sensitivity analyses rather than additional confirmatory hypotheses. To control family-wise error across the two formal comparisons, Holm-adjusted *p* values were reported [50]. The paired absolute-error comparison was also checked with an exact $2^{20}$ sign-flip randomization test. The Steiger test addresses only the specific contrast between the two dependent correlation coefficients; separate confidence-interval overlap was not used as a test of their difference. Exact *p* values, effect sizes, confidence intervals, and sensitivity results were emphasized.

Two additional analyses were added for the small sample and the direct scale comparison. First, participant influence was evaluated by deleting one participant at a time and recomputing $r_{\mathrm{MDF},Y}$, $r_{\mathrm{MNF},Y}$, and Δr for each of the 20 reduced datasets. Second, because the uncalibrated FI percentages and $Y_{p}$ need not have identical numerical calibration, a leave-one-participant-out cross-validation (LOOCV) analysis fitted Y=α+βFI on 19 participants and predicted the held-out participant. The procedure was repeated for all participants and separately for MDF and MNF. Cross-validated MAE, RMSE, $R^{2}$, observed–predicted correlation, mean prediction error, and maximum absolute error were reported. This analysis evaluates internal calibration to the archived $Y_{p}$ scale without using a participant to fit that participant’s prediction; it is not external validation and does not convert $Y_{p}$ into raw Q2. The participant-level script reproducing these analyses from the retained participant-level values reported in the Section 4 is included with the revised package.

## 4. Results

The participant-level analysis included all 20 participants who completed the haptic-writing task and post-task questionnaire. The Section 4 first summarizes the study characteristics and then reports participant-level MNF/MDF indices, internal calibration, comparative performance, and inferential sensitivity in that order. Raw Q2 questionnaire outcomes are reported separately in Section 4.7; the seven-level projection is used only as a numerical-resolution sensitivity analysis.

### 4.1. Participants and Task Completion

The study included 20 participants (12 men and 8 women) between 25 and 59 years of age. All participants completed the 400 s repetitive haptic-writing exposure and the post-task questionnaire, with no withdrawals or exclusions. Sample characteristics are summarized in Table 7.

### 4.2. Movement and Haptic-Force Characteristics

**Figure 5:**
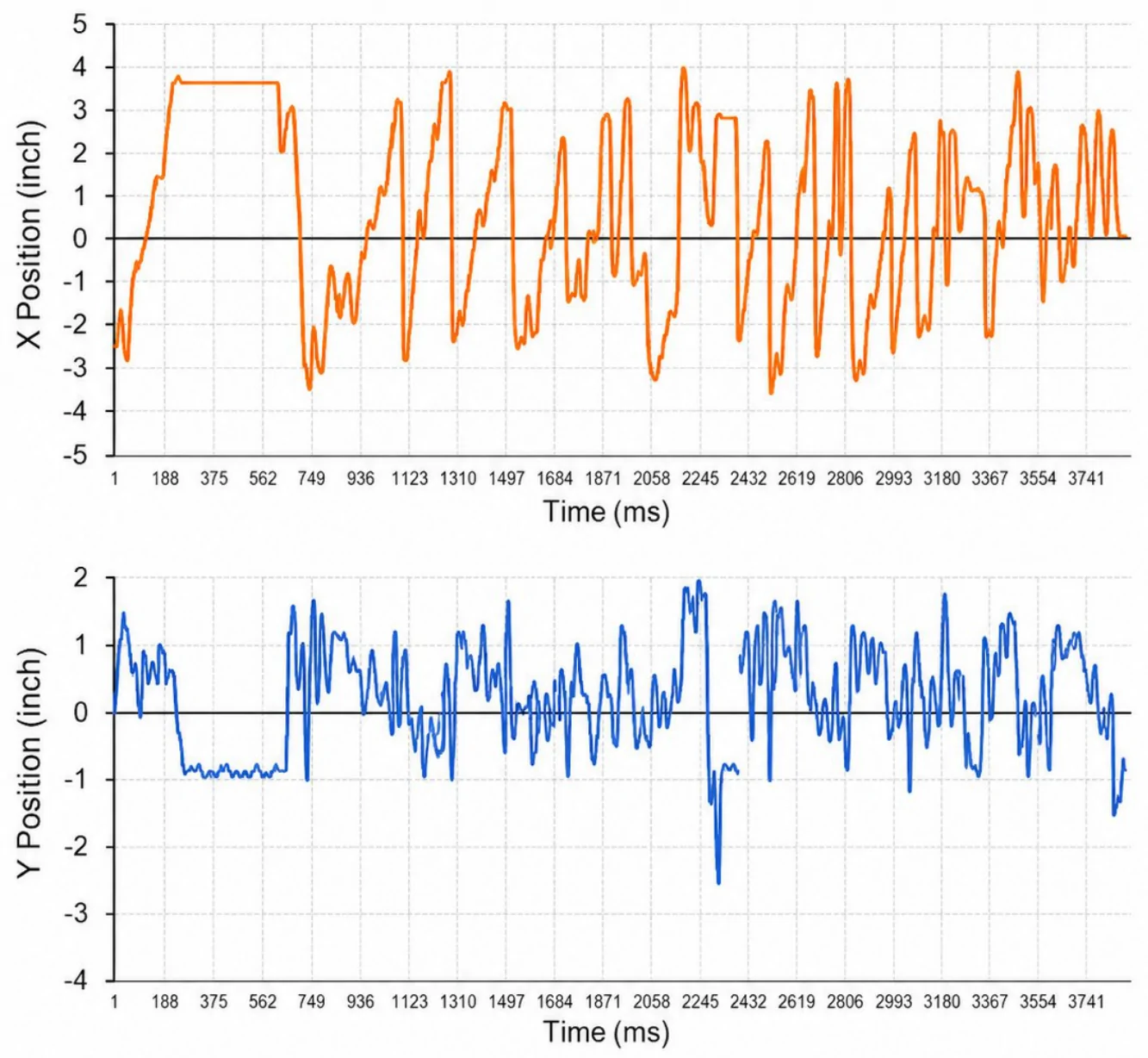

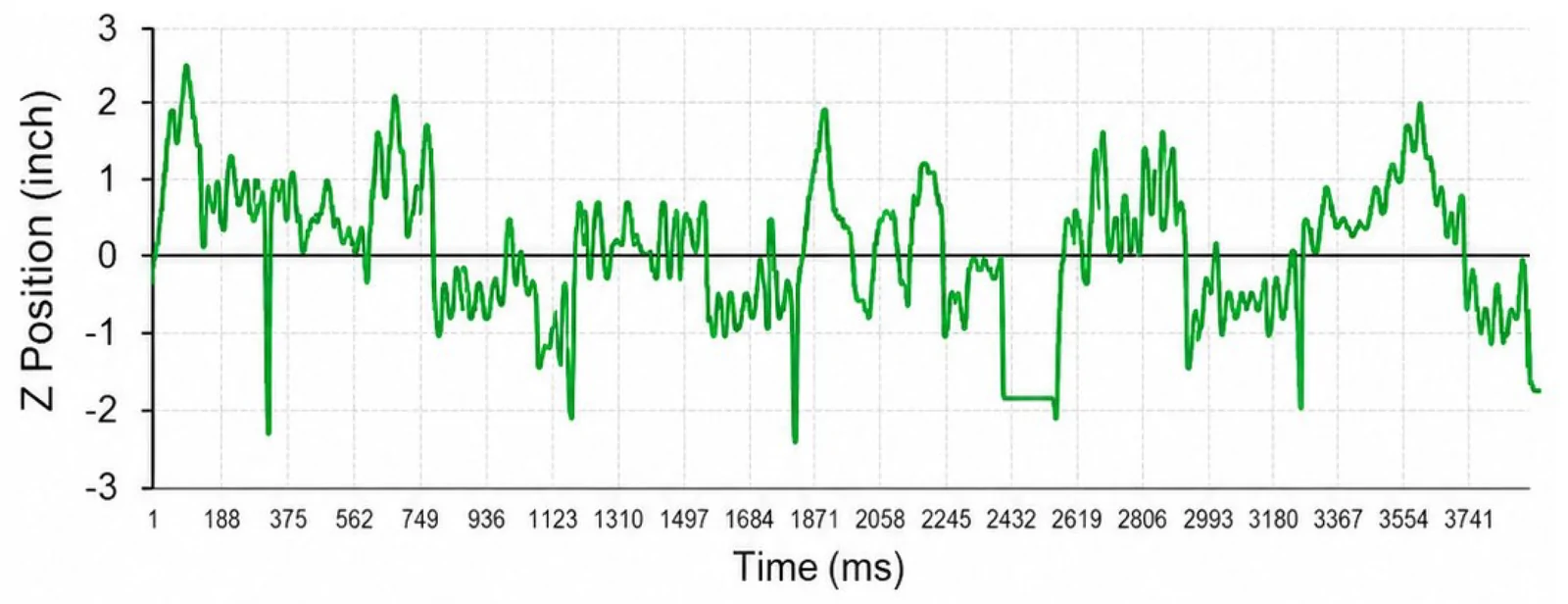
Representative three-dimensional stylus-position trajectories showing repeated displacement and direction reversals. The black horizontal reference line denotes zero position. The displayed traces are short illustrative excerpts from one participant and characterize the dynamic task exposure.

**Figure 6:**
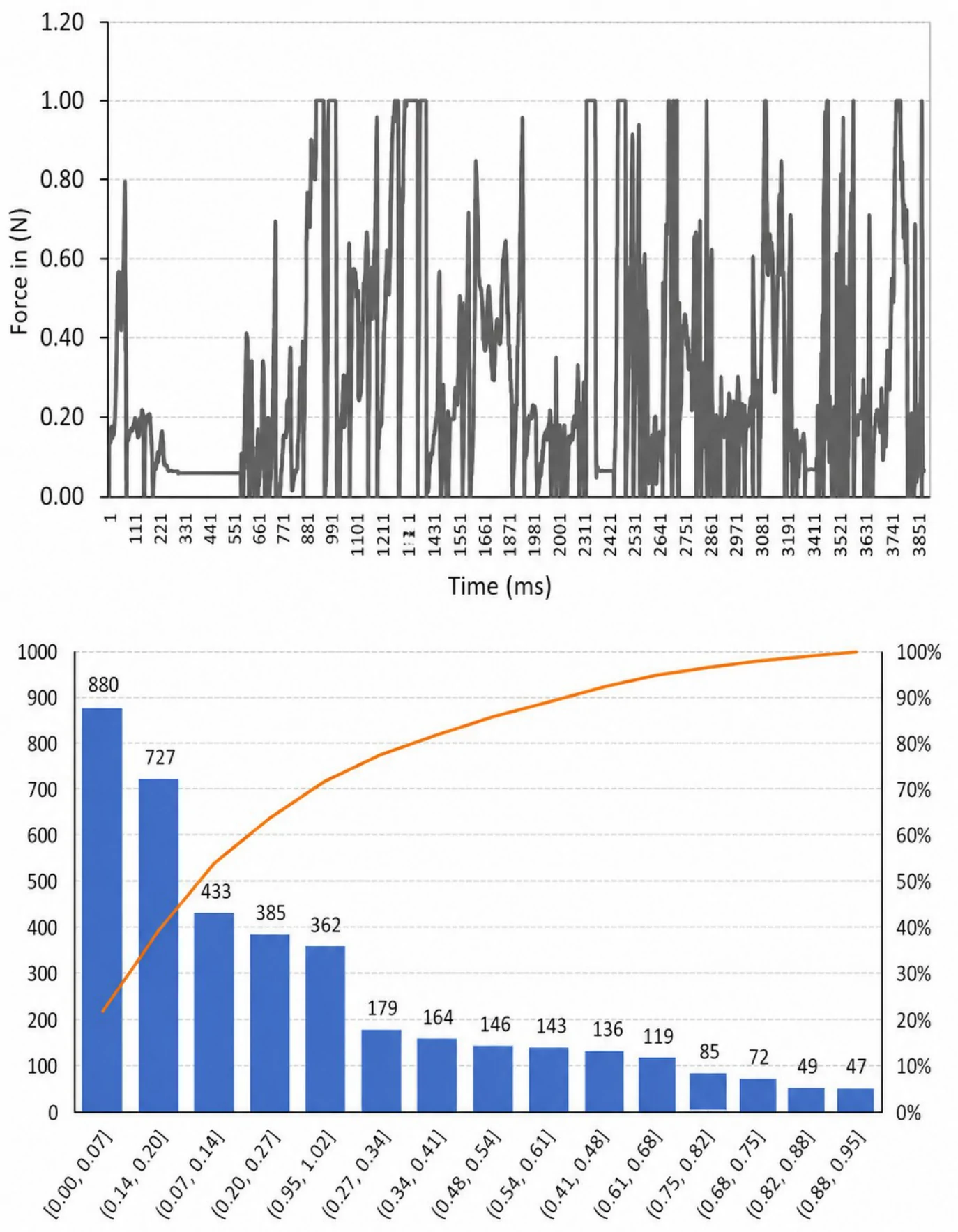
Representative interaction-force profile and corresponding Pareto distribution of force samples. The gray trace shows interaction force over time, blue bars show force-bin counts, and the orange line shows cumulative percentage. Low-force intervals were common, with intermittent peaks approaching the observed maximum. The time trace is a short illustrative excerpt from the 400 s recording.

The representative position traces (Figure 5) showed repeated displacement and direction reversals in all three axes, confirming that the protocol involved continuous movement through the haptic workspace rather than a sustained isometric contraction. The resultant-force trace and its distribution (Figure 6) were intermittent and strongly skewed: low-force intervals were common, but repeated high-force contacts were also present. High-force peaks occurred throughout the displayed recording rather than being confined to its early portion, so the illustrative trace did not show a simple monotonic loss of force. Together, the profiles show that participants experienced changing posture and contraction intensity during the task, which is important when interpreting dynamic sEMG spectral behavior.

### 4.3. Representative Physiological and Frequency-Domain Behavior

**Figure 7:**
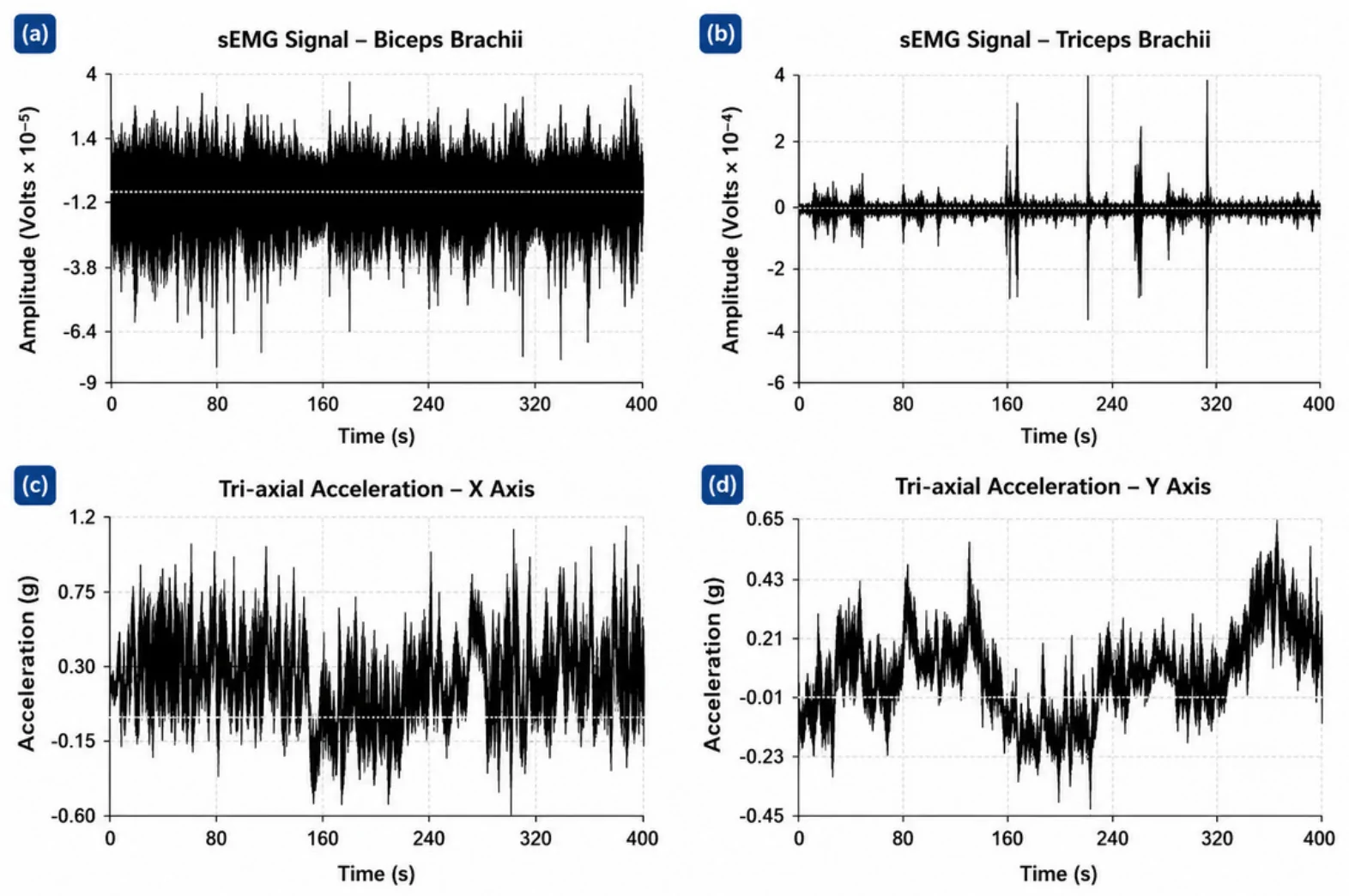
Representative synchronized recordings over the 400 s task: (**a**) biceps-brachii sEMG, (**b**) triceps-brachii sEMG, (**c**) accelerometer *x* component, and (**d**) accelerometer *y* component. The traces illustrate muscle-specific activation and nonstationary movement during dynamic stylus manipulation; horizontal dashed reference lines denote the zero-amplitude/zero-acceleration baseline.

Figure 7 presents representative sEMG recordings from the biceps and triceps brachii together with synchronized acceleration signals. Signal amplitude and temporal structure differed across channels, and the acceleration traces confirmed nonstationary movement throughout the trial. These observations support a multimuscle analysis and illustrate why raw amplitude alone was not treated as a direct measure of fatigue.

**Figure 8:**
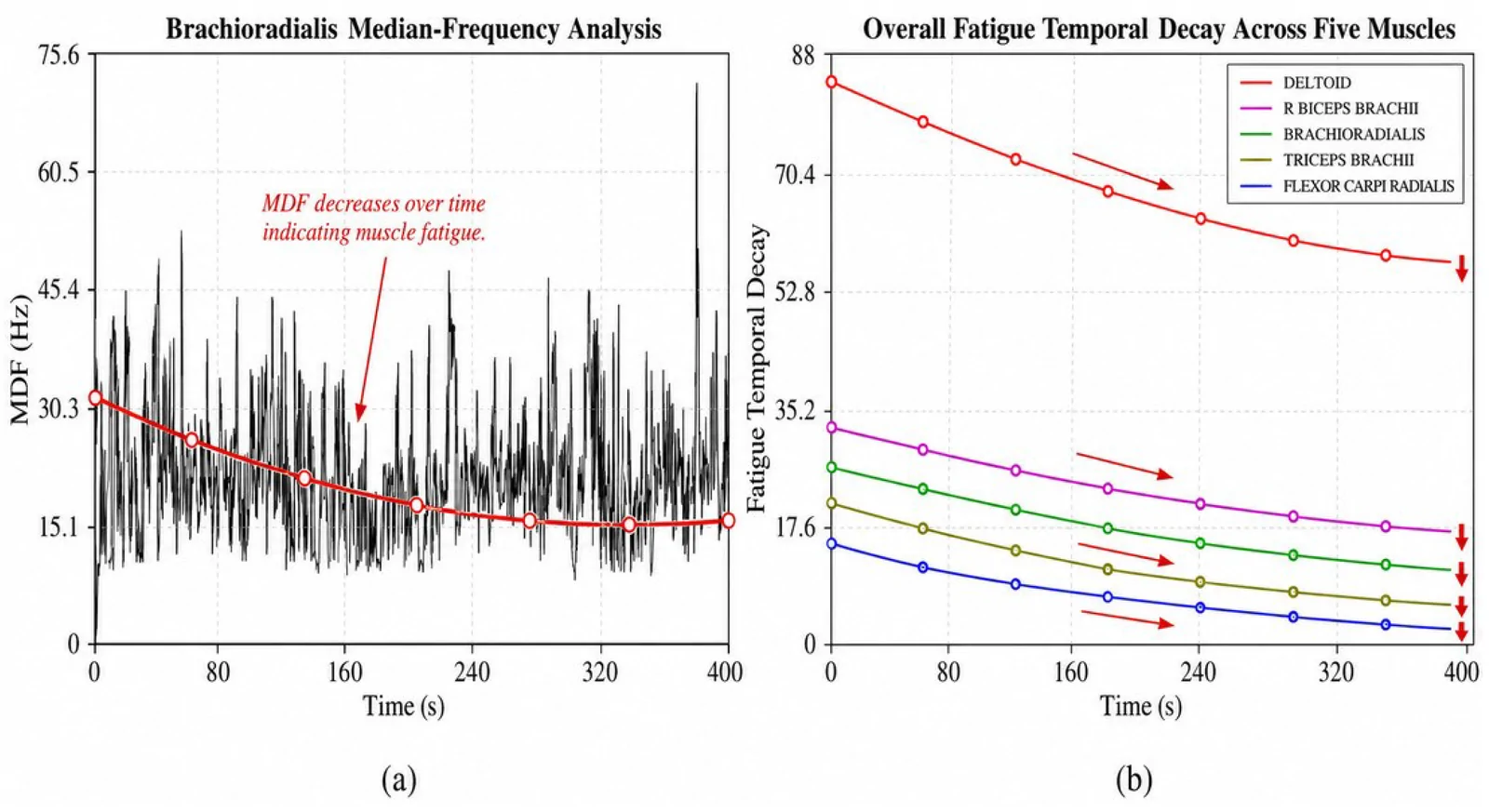
Representative median-frequency behavior for one participant (N=1). (**a**) Brachioradialis MDF estimates with the fitted temporal trajectory; (**b**) fitted MDF trajectories for the five monitored muscles from the same participant. The red arrows indicate the downward temporal direction of the fitted trajectories. The downward trends are consistent with a shift of sEMG spectral power toward lower frequencies but are not group-level summaries. The ordinate in panel (**b**), labeled “Fatigue Temporal Decay” in the archived artwork, represents the fitted MDF trajectory values in hertz. The prospective replication plan in Table 2 prespecifies cohort-level per-muscle decline, fit-quality, direction, and window-retention summaries.

For one representative participant (N=1), the MDF analysis showed downward temporal trajectories in all five monitored muscles (Figure 8). The absolute level, rate of decline, and curvature differed among the flexor carpi radialis, brachioradialis, triceps brachii, biceps brachii, and deltoid. The brachioradialis example included a late plateau, illustrating that the endpoint-based FI can summarize a net decline even when the within-task trajectory is not uniformly decreasing. The consistent direction of the fitted curves is compatible with a redistribution of sEMG spectral power toward lower frequencies during the repetitive task. The figure is used only to illustrate within-record temporal behavior. Cohort-level inference is based on the N=20 arm indices, each of which aggregates all five monitored muscles. Accordingly, the study objective and discussion have been aligned to “representative temporal behavior plus cohort-level multimuscle arm indices” rather than a claim that each individual muscle showed a uniform group-level decline. The prospective replication specification in Table 2 now prespecifies per-muscle group reporting (decline, direction, fit quality, and retained-window counts) so that this question can be tested directly in a future raw-signal analysis.

### 4.4. Participant-Level MNF and MDF Indices

The arm-level MNF index ranged from 40.10% to 93.18%, with a mean of 57.56% (SD 14.54%; 95% CI 50.75–64.36%). The MDF index ranged from 21.00% to 81.87%, with a mean of 50.62% (SD 17.60%; 95% CI 42.38–58.86%). Median values were 51.62% for MNF and 46.88% for MDF. The interquartile ranges were 48.15–69.96% for MNF and 38.50–66.08% for MDF. The values 93.18% (MNF) and 81.87% (MDF) are participant-level maxima for F8, not cohort means. Because the archived arm-level table does not retain the five channel-specific fitted initial/final frequencies used to construct those averages, the maxima cannot be decomposed retrospectively into channel denominators or fitted endpoints. The channel-plausibility audit prespecified in Table 4 is therefore required in the confirmatory raw-signal replication. The archived $Y_{p}$ fatigue-score variable ranged from 25% to 90%, with a mean of 56.95% (SD 18.23%; 95% CI 48.42–65.48%). Participant-level values are presented in Table 8.

### 4.5. Exploratory Sex-Stratified Description

Because the archived demographic field distinguished men and women but did not separately record gender identity, the subgroup summary is descriptive rather than confirmatory. Women (n = 8) showed higher mean values for the MNF-derived FI (68.18% versus 50.48%), the MDF-derived FI (61.51% versus 43.36%), and archived $Y_{p}$ scores (66.88% versus 50.33%) than men (n=12). The MDF–$Y_{p}$ association was high in both subgroups (women, r=0.968; men, r=0.929). The MNF–$Y_{p}$ association was high in women (r=0.965) but lower in men (r=0.534). Because the subgroup sizes were small and unequal and the task was not normalized to MVC, anthropometry, grip strength, or participant-level force exposure, these values are interpreted only as exploratory evidence that sex/gender-related factors should be prespecified in future studies.

### 4.6. Association, Participant Influence, Calibration, and Scale Correspondence

The MDF-derived index had the higher linear association with the archived $Y_{p}$ score (r=0.954, 95% CI 0.885–0.982, p<0.001) compared with the MNF-derived index (r=0.783, 95% CI 0.521–0.910, p<0.001). The indices were themselves correlated ($r_{\mathrm{MDF},\mathrm{MNF}}=0.786$). Their correlation difference was Δr=0.171; a direct dependent-correlation comparison yielded Steiger z=3.13 (p=0.0017; Holm-adjusted p=0.0035). The Zou 95% confidence interval for Δr was 0.055–0.423, and the participant-bootstrap 95% interval was 0.041–0.373. The paired label-swap randomization sensitivity test was less conclusive (p=0.082). The corresponding coefficients of determination were 0.910 and 0.613, and Spearman correlations showed the same ordering (ρ=0.933 for MDF and ρ=0.677 for MNF).

The leave-one-participant-out influence analysis showed that this ordering was not attributable to a single influential participant. Across the 20 reduced datasets, MDF correlations ranged from 0.944 to 0.978, MNF correlations ranged from 0.726 to 0.820, and Δr remained positive in 20/20 analyses, ranging from 0.125 to 0.218. Thus, the within-sample MDF > MNF ordering was stable to participant deletion even though the exact paired label-swap test did not reach the conventional 0.05 threshold. The combined evidence identifies MDF as the more promising task-specific candidate for prospective validation in this protocol; it does not establish universal feature superiority.

In the seven-level discretization stress test, each archived $Y_{p}$ value was projected to the nearest numerical level defined by Equation (25); this is not a reconstruction of the raw Q2 responses. The relative ordering was unchanged. Pearson correlation remained higher for MDF (r=0.908, 95% CI 0.778–0.963, p<0.001) than for MNF (r=0.722, 95% CI 0.411–0.883, p<0.001); the corresponding Spearman correlations were ρ=0.892 and ρ=0.664. The dependent-correlation comparison remained positive in this sensitivity analysis (Steiger z=2.48, p=0.013), while the participant-bootstrap interval for Δr included zero (−0.007 to 0.432) and the paired label-swap randomization result was p=0.225. These secondary results show that MDF remained the higher point estimate after coarsening the archived scale, while the wider bootstrap uncertainty and label-swap result (p=0.225) show that the discretized sensitivity analysis is supportive of direction consistency rather than a separate confirmatory superiority test. These discretized-score results are reported separately from the archived-scale estimates in the comparative-performance table below.

Before calibration, MDF produced lower participant-level numerical error: MAE was 6.49 percentage points and RMSE was 8.28 percentage points, compared with 8.59 and 11.08 percentage points for MNF. MAPE was 11.92% for MDF and 19.18% for MNF. The mean difference in absolute error was 2.10 percentage points; this paired difference was not statistically significant (p=0.274; Holm-adjusted p=0.274), the exact sign-flip sensitivity test gave p=0.281, and the bootstrap 95% interval was −1.32 to 5.81 percentage points. These quantities are therefore interpreted as uncalibrated scale correspondence rather than as a formal prediction comparison.

Because raw FI percentages and $Y_{p}$ are not validated as commensurate measurement scales, the following raw difference analysis is retained only as an exploratory scale-correspondence diagnostic, not as evidence that the two methods measure an interchangeable physiological quantity. The diagnostic identified different error structures on the archived $Y_{p}$ scale. MNF had little mean bias relative to $Y_{p}$ (0.61 percentage points), but wide conventional 95% limits of agreement (−21.63 to 22.85 percentage points). MDF had narrower conventional limits (−17.05 to 4.39 percentage points) but a mean bias of −6.33 percentage points, indicating systematic underestimation. Shapiro–Wilk diagnostics did not indicate marked non-normality for MNF differences (p=0.410), whereas MDF differences departed from normality (p=0.019). Regressions of difference on paired mean did not identify proportional bias (MNF slope p=0.137; MDF slope p=0.626), although power was limited. Empirical 2.5th–97.5th percentile intervals were −18.95 to 23.12 for MNF and −19.06 to 0.37 for MDF. Under the discretization stress test criterion, bias and conventional limits were 1.72 (−23.04 to 26.49) for MNF and −5.22 (−20.29 to 9.86) for MDF. These diagnostics show the agreement pattern on the archived $Y_{p}$ scale. They also demonstrate why correlation must not be interpreted as numerical agreement: the MDF Pearson correlation was r=0.954 (with $R^{2}=0.910$), yet MDF showed a mean bias of −6.33 percentage points and non-normal differences. Neither *r* nor $R^{2}$ implies numerical interchangeability.

The LOOCV calibration analysis addressed a different question: whether each physiological index could be mapped to the archived $Y_{p}$ scale using only the other participants. MDF achieved cross-validated MAE 4.38 percentage points, RMSE 5.87, $R^{2}=0.891$, and observed–predicted r=0.944, compared with MNF MAE 9.24, RMSE 12.02, $R^{2}=0.542$, and r=0.739. Mean prediction error was close to zero for both models (−0.06 for MDF and −0.10 for MNF), while maximum absolute error was 17.97 and 27.87 percentage points, respectively. On this calibrated held-out scale, conventional difference limits were −11.87 to 11.75 percentage points for MDF and −24.28 to 24.08 for MNF; empirical 2.5th–97.5th percentile intervals were −14.29 to 7.13 and −21.67 to 24.14, respectively. MDF held-out residuals remained non-normal (Shapiro–Wilk p=0.019), so the empirical interval is emphasized. These held-out internal results strengthen the task-specific case for MDF because no participant contributed to the calibration model used for that participant’s prediction; nevertheless, they remain internal calibration to $Y_{p}$, not external validation or reconstruction of raw Q2.

Table 9 summarizes the exploratory leave-one-participant-out internal-calibration results.

Table 10 summarizes the association, discretization-sensitivity, and uncalibrated scale-correspondence results.

Table 11 summarizes the inferential hierarchy, multiplicity handling, and resampling sensitivity analyses.

**Figure 9:**
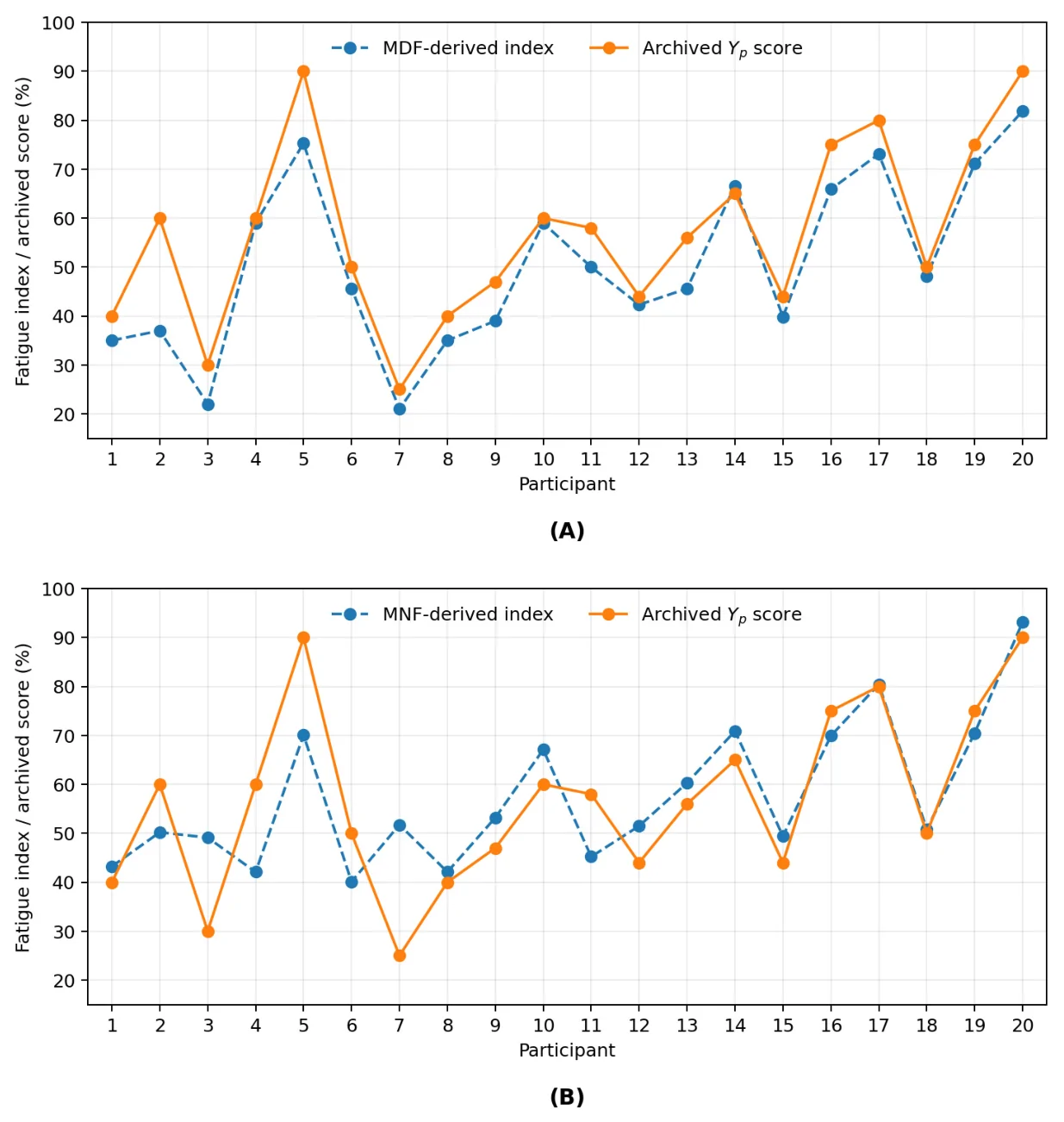
Participant-level comparison of the archived 0–100 analysis criterion $Y_{p}$ with the sEMG-derived arm-fatigue indices, reconstructed directly from the participant-level values in Table 8. Each panel contains the same N=20 participants: (**A**) MDF-derived index and (**B**) MNF-derived index. Raw Q2 questionnaire outcomes are reported separately in Figure 10 and Table 6.

Figure 9 displays all 20 participants in each panel; unlike the representative signal and trajectory figures, it is a participant-level group comparison.

### 4.7. Post-Task Questionnaire Outcomes

**Figure 10:**
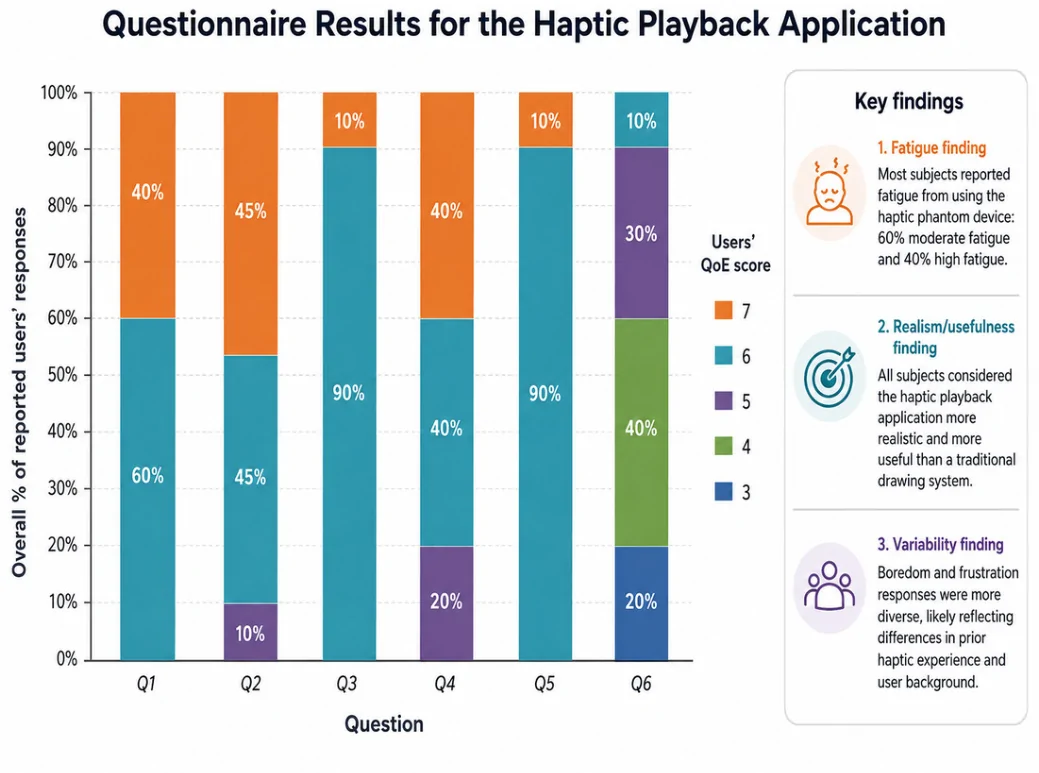
Questionnaire results for the haptic playback application. Each Q1–Q6 stacked bar summarizes the same N=20 participants and shows the percentage distribution of seven-point ratings. The summary panel highlights the fatigue response, favorable realism/usefulness ratings, and greater variability in boredom/frustration-related responses. For Q2, the recorded categories are 5, 6, and 7; these questionnaire counts are reported separately from the archived participant-level $Y_{p}$ analysis criterion, with the numerical comparison shown in Table 6.

The post-task questionnaire results are summarized in Figure 10; each item includes all N=20 participants. The fatigue-related items indicate that the task was physically demanding: for Q1, 60% of participants selected a score of 6 and 40% selected a score of 7; for Q2, responses were distributed across scores 5 (10%), 6 (45%), and 7 (45%). These distributions are consistent with moderate-to-high perceived fatigue after the 400 s haptic-writing exposure. Q2 therefore had a mean of 6.35/7 (89.17/100 under direct linear rescaling). Table 6 shows that these recorded categories differ from the distribution obtained by discretizing $Y_{p}$, so the questionnaire and archived analysis scale are reported as separate outcomes rather than forced into a single transformation.

The non-fatigue QoE items were generally favorable. Q3 was dominated by a score of 6 (90%), with the remaining 10% at 7; Q5 showed the same 90%/10% split. Q4 remained positive but was more dispersed, with 20% at score 5, 40% at 6, and 40% at 7. These patterns indicate that participants generally regarded the haptic playback application as realistic and useful even though it produced noticeable arm fatigue.

The greatest variability occurred for Q6, with responses spanning scores 3 (20%), 4 (40%), 5 (30%), and 6 (10%). This wider distribution suggests that boredom and frustration were more participant-dependent than the fatigue and usefulness judgments, potentially reflecting differences in previous haptic experience, tolerance for repetitive work, or expectations about the interface. These secondary QoE outcomes were interpreted separately from the archived $Y_{p}$ comparison and were not combined with the arm-fatigue score. Taken together, the questionnaire results reveal an important trade-off: favorable judgments of realism and usefulness coexisted with substantial perceived fatigue. This pattern motivates pairing application-level QoE measures with physiological workload measures during haptic-system evaluation.

## 5. Discussion

This study investigated whether frequency-domain features of multimuscle sEMG can characterize upper-limb fatigue during a repetitive haptic-writing task. Four findings are most relevant. First, the cohort-level arm indices summarize five-muscle spectral change, while the detailed five-muscle trajectory figure is explicitly representative (N=1). Second, MDF showed the higher association with the archived participant-level analysis score. Third, this MDF > MNF ordering remained positive after deleting each participant in turn, which addresses the concern that the result might be driven by one influential observation, although the exact label-swap sensitivity test remained inconclusive. Fourth, simple calibration trained on the other 19 participants produced lower held-out MAE and RMSE for MDF in this cohort. Together, these analyses identify MDF as the more promising task-specific candidate for prospective validation in this dynamic haptic protocol, while raw Q2 descriptives and the archived $Y_{p}$ analysis scale remain analytically separate.

The findings are consistent with, and add a specific haptic case to, the recent dynamic-fatigue literature. Cerqueira et al. paired upper-limb sEMG with self-perceived fatigue across multiple dynamic movements, establishing the value of participant-reported fatigue labels outside isometric protocols [27]. Mathew et al. emphasized heterogeneity in wearable upper-limb fatigue methods and the need to keep feature direction and task definition explicit [28]. Coraggio et al. directly compared multiple linear sEMG fatigue algorithms during repetitive upper-limb movements and showed that relative algorithm performance is task- and feature-dependent rather than universal [29]. Daniel et al. further highlighted posture, contraction intensity, electrode behavior, and preprocessing as central challenges in dynamic sEMG fatigue assessment [41]. Against that background, the present contribution is a task-specific, five-muscle force–feedback comparison that adds dependent-correlation inference, participant-deletion influence analysis, and held-out internal calibration. Surgical and rehabilitation studies provide the translational context by showing that muscle fatigue remains measurable during robot-assisted and haptic work even when assistance improves other ergonomic outcomes [4,5,6,7,8,37].

### 5.1. Physiological Interpretation of the Spectral Changes

The observed shift of the sEMG spectrum toward lower frequencies is consistent with established myoelectric manifestations of localized muscle fatigue. During sustained or repeated contractions, reductions in muscle-fiber conduction velocity, altered motor-unit recruitment, and changes in motor-unit synchronization can contribute to decreases in spectral-frequency measures [22,23,32]. The haptic-writing task produced repeated cycles of stylus motion, force exchange, and upper-limb stabilization, allowing the spectral analysis to describe fatigue-related change during a dynamic interaction rather than a static contraction.

MNF and MDF are informative descriptors in this setting because the analysis targets slow, directional change over a 400 s exposure rather than instantaneous frequency differences between individual movements. Short overlapping windows reduce within-window non-stationarity, repeated writing cycles sample a broad range of postures and force states, and the fitted start-to-end trajectory reduces dependence on any single movement phase. The recurrence of downward trends across muscles acting at different joints is consistent with a distributed fatigue-related response. The synchronized force, position, and acceleration recordings provide mechanical context for interpreting the sEMG trajectories during continuous haptic interaction.

The quadratic model uses the complete accepted trajectory, and Equation (18) summarizes that trajectory as a single net start-to-end change. Curvature, turning points, monotonicity, and residual fit describe complementary aspects of temporal behavior, whereas the participant-level analysis focuses on the quadratic endpoint index.

The involvement of five muscles is physiologically plausible for the task. The flexor carpi radialis and brachioradialis contribute to wrist and forearm control, the biceps and triceps stabilize and move the elbow, and the deltoid contributes to shoulder positioning. In the representative record, downward trends across the five channels were consistent with distributed demand across several levels of the upper limb. This multimuscle perspective is an advantage over a single-channel estimate because fatigue during human–device interaction may shift among muscles as participants modify posture or movement strategy. Equal averaging provides a transparent way to summarize the distributed upper-limb response across the five monitored muscles.

### 5.2. Task-Specific Comparison of MDF and MNF

MNF and MDF summarize different properties of the power spectrum. MNF is a power-weighted centroid and is therefore influenced by the magnitude and location of spectral extremes, whereas MDF is defined by the frequency that divides cumulative spectral power into two equal regions [24]. In this dataset MDF had the higher association (Δr=0.171), the ordering remained positive in every leave-one-participant-out analysis, and MDF retained substantially lower held-out error after simple LOOCV calibration. These convergent within-sample results make MDF the more promising task-specific candidate for the next prospective validation study. The exact label-swap test (p=0.082) and the discretized-scale sensitivity analysis prevent a stronger claim that MDF is universally superior. This interpretation is also consistent with Coraggio et al., who found that the relative performance of sEMG fatigue algorithms varies with movement and feature definition [29]. A plausible explanation for the task-specific MDF ordering is its cumulative half-power definition, which can be less affected than the spectral centroid by isolated high- or low-frequency power. That mechanism should be tested directly in a prospectively specified raw-signal replication using the bandwidth and preprocessing sensitivities in Table 2.

The revised analysis separates three questions that were previously conflated: association, uncalibrated scale correspondence, and calibration. Correlation evaluates co-variation with the archived score. The raw Bland–Altman comparison evaluates what happens if the FI percentage is placed directly on the $Y_{p}$ scale without calibration; under that comparison MDF showed a −6.33 percentage-point offset and non-normal differences, while MNF had little average offset but much wider dispersion. The LOOCV analysis then asks whether a simple mapping learned from other participants can calibrate each physiological index. MDF produced lower held-out internal-calibration error (MAE 4.38; RMSE 5.87; $R^{2}=0.891$) than MNF (MAE 9.24; RMSE 12.02; $R^{2}=0.542$) in this cohort. Thus, the high MDF correlation is not treated as one-to-one agreement; rather, it identifies a physiological signal with promising internal calibratability that now requires prospective criterion collection and external validation.

### 5.3. Sex/Gender Dimension

The exploratory subgroup description suggested higher average archived $Y_{p}$ values and higher MNF/MDF arm indices in women than in men, while the MDF–$Y_{p}$ association remained high in both subgroups. This pattern should not be interpreted as a biological sex effect because the sample was small, the groups were unequal, and the task load was not normalized to MVC, arm anthropometry, grip strength, or individualized force exposure. Nevertheless, the observation is consistent with the broader ergonomic concern that identical hand-intensive tasks can impose different relative muscular demands across sexes. Dahlgren et al. found similar wrist angular velocities but substantially higher normalized forearm muscle activity and less muscle-recovery time in women performing identical hand-intensive work tasks [51]. Other dynamic-fatigue research also shows that sex-related differences in fatigability depend on task mechanics and fatigue definition [52]. Future haptic-fatigue studies should therefore balance recruitment, record sex and gender variables separately where appropriate, collect strength or MVC reference measures, and prespecify subgroup or interaction analyses rather than relying on post hoc comparisons.

### 5.4. Robustness to Analysis Choices and Task Mechanics

The reviewer-requested robustness checks now have an explicit status in Table 4. Two analyses were actually conducted from the retained participant-level arm-index table: leave-one-participant-out influence analysis and leave-one-participant-out internal calibration. Muscle-level distributions, leave-one-muscle-out aggregation, longer-window sensitivity, alternative trajectory fitting, mechanical covariate adjustment, and channel-level plausibility checks were not conducted because they require participant-linked window-, muscle-, QC-, or mechanical-level records that are not retained. They are therefore prespecified for prospective raw-signal replication rather than presented as completed robustness evidence. This distinction is important in dynamic sEMG because contraction intensity, muscle length, posture, and movement can alter spectral features independently of fatigue [33].

The 8 Hz native bin spacing is likewise treated as an analysis-design limitation rather than being hidden by interpolation. Within-bin MDF interpolation makes the crossing estimate continuous, but it does not create the spectral information of a longer window. A raw-signal replication should therefore repeat the analysis with 250- and 500 ms windows and compare participant-level indices and the MDF-MNF ordering. The quadratic trajectory should also be compared with a linear fit and a robust smooth trend. Chand et al.’s dynamic human–robot study further supports joint consideration of sEMG and task load when estimating fatigue during repetitive collaborative work [30].

Equal five-muscle averaging is retained as a transparent primary aggregation because it is independent of the outcome variable, but it is not presented as biomechanically optimal. The confirmatory analysis consequently requires five leave-one-muscle-out recomputations and, where an independent normalization contraction is available, an activation-weighted sensitivity analysis. Participant-linked mean force, force variability, movement amplitude/path length, and RMS acceleration should similarly enter parsimonious covariate models. Persistence of the MDF association after these adjustments would strengthen a fatigue interpretation; material attenuation would indicate that task mechanics explain part of the observed spectral shift.

### 5.5. Implications for Health 5.0 Medical Robotics and Human-Centric Industry 5.0

The Health 5.0 discussion is translational rather than experimental: the present protocol used a general-purpose haptic stylus with healthy adults and did not evaluate a clinical robot, patient intervention, surgical procedure, or adaptive controller. Health 5.0 frames physiological-state awareness as part of person-centered medical robotics. In robot-assisted surgery, the person physically coupled to the system is primarily the surgeon or teleoperator. Console-based control can reduce some whole-body ergonomic burdens relative to conventional laparoscopy, yet prolonged procedures still involve static posture, repeated hand movements, visual concentration, and muscle-specific loading [7,8]. Candidate inputs for fatigue-aware surgical training and teleoperation include sEMG spectral trends, grip force, master-handle kinematics, tool–tissue force, task error, and procedure phase. Candidate responses include micro-break alerts, console-support or haptic-gain adjustment, subtask redistribution, and flags for deteriorating motor control. These responses would need to remain aligned with surgical safety, clinician authority, and the need to minimize distraction during critical phases.

In robot-assisted rehabilitation, the person physically coupled to the robot is usually the patient, although therapist fatigue can also matter in shared-control or bilateral tele-rehabilitation. Repetition and resistance are needed to drive motor learning, but excessive fatigue may degrade movement quality, encourage compensatory strategies, or terminate the session prematurely. Prior robot-assisted training research shows that fatigue changes EMG features and their relation to interaction force, while recent systems have begun to incorporate EMG-based fatigue checks into adaptive rehabilitation protocols [4,5]. A prospectively validated multimuscle index represents a potential input to patient-specific adjustment of assistance, resistance, workspace, repetition count, or rest. Such an index remains complementary to therapist observation, patient-reported exertion, pain monitoring, and functional performance.

The present results support continued investigation of the sensing component of this vision by showing a high within-sample association between an arm-level MDF summary and the archived fatigue-score variable during repetitive haptic interaction; they do not establish agreement with, or numerical replacement of, the raw Q2 questionnaire. In medical-robot settings, multimuscle spectral features could be combined with handle kinematics, interaction forces, task phase, and performance measures to describe the user’s physiological state during training, telemanipulation, or rehabilitation tasks. These data streams provide a foundation for person-centered monitoring that remains aligned with clinician oversight and task-specific safety requirements.

Industry 5.0 presents a parallel context in collaborative manufacturing, remote inspection, training, and maintenance. Haptic and immersive interfaces may enhance perception and dexterity, while excessive repetitive load conflicts with their human-centric purpose. Multimuscle surface electromyography can serve as a possible input channel to the human-in-the-loop supervisory layer, which controls assistance, resistance, task delegation, pacing, or rest scheduling. In the context of the Health 5.0 and Industry 5.0 paradigms, human capabilities, autonomy, and well-being become design criteria alongside task execution efficiency.

Recent advances in haptics make such consideration highly relevant. Energy-harvesting interfaces can deliberately introduce resistance to generate energy from user movement [9], and shared haptic feedback can assist with fast, repetitive collaborative tasks in the VR environment [3]. The haptic feedback provided by medical robots can enhance the perception of interaction forces and guidance, though prolonged use, untuned resistance, or an improper console position can add an extra physical load. The idea of a fatigue-aware controller corresponds to adaptation of the magnitude of force feedback, assistance, control gain, working space, repetition rate, or rest schedule depending on the user’s neuromuscular condition.

The fatigue-monitoring perspective complements the haptic fatigue-mitigation approach. The [26] paper considers the effects of combined force and electrical stimulation after fatigue induced by exercise and demonstrates recovery-related changes in the frequency domain of the sEMG signal. This work focuses on detecting fatigue during task performance. Detection and mitigation are the two complementary functionalities in Health 5.0 haptic systems. Decreasing the haptic force may affect surgical accuracy or the intensity of rehabilitation, whereas electrical stimulation adds an additional physiological effect.

### 5.6. Methodological Strengths

Several features strengthen the study. The task was an ecologically relevant, dynamic haptic interaction rather than a purely isometric laboratory contraction. Five muscles were monitored simultaneously, allowing the arm index to represent wrist, elbow, and shoulder involvement. Force, three-dimensional position, acceleration, sEMG, and subjective ratings were collected within the same experimental session. The study also compared two commonly used spectral measures instead of assuming their equivalence. The revision further adds two analyses that are especially informative for a small proof-of-concept cohort: leave-one-participant-out influence analysis to test result stability and LOOCV calibration to evaluate held-out internal performance. The raw Q2 distribution and archived $Y_{p}$ analysis variable are also separated numerically rather than treated as interchangeable scales.

### 5.7. Limitations

Several limitations qualify these findings. First, the modest sample (n=20), single laboratory setting, single task, and single exposure duration limit subgroup analysis and generalization across haptic contexts. No a priori sample-size calculation was documented, so the cohort should be regarded as a fixed pilot/proof-of-concept sample rather than a powered validation cohort. The absence of a non-haptic, low-force, or rest-control condition also limits the separation of haptic force from repetition, posture, visual attention, and task duration. The sample also prevents robust sex/gender inference; the subgroup description was post hoc and not adjusted for strength, anthropometry, or force exposure.

Second, self-paced execution and the absence of MVC normalization limit the separation of fatigue-related spectral change from variation in contraction intensity. Force, posture, movement artifact, and changes in the electrode–muscle relationship may have contributed to the spectral trajectories. The results therefore characterize application-level spectral change rather than a contraction-normalized physiological measure.

Third, raw Q2 and the archived $Y_{p}$ variable are distinct numerical data products. The provenance diagnostic now demonstrates that $Y_{p}$ is not a direct linear recoding of the recorded Q2 distribution; consequently, raw Q2 is used for questionnaire descriptives and $Y_{p}$ is used only for the legacy participant-level analysis scale.

Fourth, the retained historical record does not contain every raw-signal preprocessing parameter or participant-by-muscle QC count. The numerical Butterworth/Bessel settings, 20–400/20–450 Hz bands, QC thresholds, and reprocessing sensitivities in Table 2 are prospective recommendations only and were not used to generate the reported historical results. The revised package includes executable code only for the participant-level influence and internal-calibration analyses that can be reproduced from Table 8.

Fifth, the available source package supports cohort-level arm indices but not the participant-by-muscle/window matrix required for per-muscle cohort summaries, leave-one-muscle-out aggregation, window-length/model sensitivity, or participant-level mechanical covariate adjustment. Therefore Figure 8 is explicitly illustrative and the study’s cohort-level claims are restricted to the five-muscle aggregated arm indices. Table 4 converts these data limitations into a prespecified confirmatory analysis set rather than assuming invariance to preprocessing, aggregation, or task mechanics.

Sixth, the findings remain proof-of-concept evidence from a study-specific task and have not been externally validated.

### 5.8. Future Research

Future work should prospectively freeze the preprocessing specification in Table 2, archive executable code and QC logs, and determine the confirmatory sample size from a prespecified precision target or minimally important MDF-MNF difference rather than from the pilot *p* value. The next study should also preserve participant-linked raw Q2 responses so that ordinal (Spearman and ordinal-regression) analyses can be performed directly, while a separate calibrated continuous fatigue criterion can be validated independently if desired. Retaining window-level trajectories would allow routine reporting of adjusted $R^{2}$, normalized residual error, derivative-based monotonicity, and interior turning points. For each muscle, future reports should provide group-level start-to-end MNF/MDF decline, the proportion of participants with positive FI, fit-quality distributions, and retained-window summaries. Mixed-effects or functional-data models could then separate within-participant temporal change from between-participant differences while preserving non-monotonic behavior that is compressed by a single endpoint index.

Prospective studies could include a lower-load or non-haptic control condition, repeated in-task fatigue ratings, standardized performance outcomes, and recovery measurements. In future iterations, a brief standardized submaximal reference contraction collected before the naturalistic haptic task could provide an individual normalization baseline without constraining the main exposure. Participant-linked force streams would permit participant-level force summaries and parsimonious force-adjusted models; persistence of the FI–$Y_{p}$ association after adjustment would reduce, although not eliminate, the contraction-intensity explanation. Validation across haptic tasks, force profiles, exposure durations, and participant populations would clarify generalizability. Future work should also report sex and gender variables separately when collected, recruit sufficient subgroup sizes, and include normalization variables needed to distinguish absolute task exposure from relative physiological demand. Equal aggregation could be compared with activation-based weighting using normalized RMS amplitude, as well as weights based on force contribution, anatomical function, or task phase. Closed-loop studies could then evaluate whether fatigue-responsive adaptation reduces physical demand without degrading accuracy, realism, learning, or user acceptance. Health 5.0 translation should proceed through distinct medical-robot studies. Surgical studies should recruit trainees and experienced clinicians, use validated console or teleoperation tasks, record master-handle and tool–tissue forces, and test whether multimuscle spectral trends predict loss of precision, tremor, errors, or the need for micro-breaks. Rehabilitation studies should recruit diagnosis-specific patient groups, quantify pain and compensatory movement, and test clinician-approved adaptation of assistance, resistance, repetition count, and rest. Shared-control and tele-rehabilitation studies should monitor both patient and clinician when both are physically coupled to the system. Across these studies, adaptation rules should be prospectively specified, safety bounded, explainable to users, and evaluated for adverse events, adherence, functional recovery, and preservation of task performance.

## 6. Conclusions

This study examined whether frequency-domain sEMG features recorded from five upper-limb muscles could characterize fatigue during a 400 s repetitive haptic-writing task. Participant-level multimuscle indices reflected net spectral decline, while representative channel trajectories illustrated lower-frequency shifts in muscles acting at the wrist, elbow, and shoulder. Within this proof-of-concept cohort, MDF emerged as the more promising spectral summary: it had the higher association with the archived analysis score, the MDF > MNF correlation difference remained positive after omission of every participant, and exploratory LOOCV calibration produced lower held-out error in this cohort. The exact label-swap test remained inconclusive, so the result is interpreted as a task-specific prioritization of MDF for prospective validation rather than universal superiority.

The principal contribution is a proof-of-concept framework that combines multichannel sEMG, movement-related measurements, and participant-reported fatigue to evaluate physiological demand during dynamic haptic interaction. The findings also demonstrate that favorable ratings of realism and usefulness can coexist with substantial perceived fatigue, showing that conventional quality-of-experience outcomes do not capture physiological workload. The revised analysis distinguishes raw Q2 questionnaire responses from the archived $Y_{p}$ analysis scale, separates uncalibrated scale correspondence from calibration, and provides an explicit prospective preprocessing specification. These changes convert the main numerical result into a clear research hypothesis: MDF is the more promising task-specific candidate for prospective validation and should be tested, calibrated, and compared with MNF against participant-linked fatigue criteria in a larger independent cohort.

The MDF-derived index is therefore a more promising task-specific candidate physiological input for future fatigue-aware haptic systems, subject to prospective preprocessing, direct criterion collection, calibration, comparison with MNF, and independent replication. These are prospective applications rather than demonstrated outcomes of the present experiment. Potential Health 5.0 applications include clinician-fatigue monitoring during console control and haptic telemanipulation, together with individualized assistance, resistance, repetition, and rest in rehabilitation robotics under patient and clinician oversight. Industry 5.0 presents a parallel application in collaborative and remote work. Together, the results motivate continued development of objective upper-limb fatigue characterization for human-centric haptic interaction.

## Figures and Tables

**Table 1:** Selected studies related to wearable fatigue sensing, haptic interaction, medical robotics, industrial ergonomics, and Health 5.0 monitoring. The table is a focused comparison rather than a systematic review.

Study	Setting and Task	Sensing or Feedback	Main Outcome	Relation to the Present Study
Caporaso et al. [2]	VR simulation of an automotive human–robot cooperative workplace	Five sEMG sensors and one accelerometer	Real-time muscle-activity-based ergonomic indices displayed in VR	Strong industrial and VR relevance; focused on activation and ergonomic scoring rather than spectral fatigue trajectories
Galofaro et al. [36]	Virtual object lifting with NMES-rendered haptic load	NMES, kinematics, metabolic measures, and perceived fatigue	NMES reproduced load-related effort and fatigue more closely than visual-only interaction	Demonstrates that haptic rendering changes physical demand; did not estimate multimuscle fatigue from sEMG spectra
Moyen-Sylvestre et al. [39]	Repetitive pointing and low-load work tasks	Eleven IMUs measuring acceleration and angular velocity	Fatigue altered movement- and tremor-band spectra in a task-dependent manner	Supports multimodal contextualization of dynamic fatigue; no sEMG-based arm index or haptic task
Merbah et al. [37]	Prolonged surgical procedure	Wearable multimuscle sEMG	Muscle activity and fatigue visualized across task phases	Shows feasibility in professional work; single-case ergonomic design and no VR/haptic interaction
Armijo et al. [7]	Laparoscopic and robot-assisted operations	Upper-limb sEMG activation and MDF plus perceived fatigue	Surgical approach produced muscle-specific activation and fatigue patterns	Direct medical-robot relevance; shows that robotic ergonomics do not eliminate localized clinician fatigue
Pérez-Salazar et al. [8]	Simulator and experimental robotic-assisted surgical procedures	sEMG, motion, ECG, EDA, force, and workload ratings	Robotic assistance improved several ergonomic measures while stress and localized loading remained measurable	Supports multimodal clinician-state monitoring in Health 5.0 surgical robotics
Poyil et al. [4]	Assist-as-needed upper-limb training with a HapticMaster robot	sEMG median frequency, average power, and robot kinematics/force	Fatigue altered EMG features and EMG–force correlation; assistance reduced fatigue for some users	Closest rehabilitation-robot analogue; examined selected muscles rather than an arm-level multimuscle index
Covaciu et al. [5]	VR-supported robot-assisted lower-limb rehabilitation	EMG-driven adaptive control using MDF and RMS fatigue checks	Protocol changed the rehabilitation workflow when fatigue-related EMG deterioration was detected	Demonstrates a Health 5.0 fatigue-aware control concept; different limb and not a validation of calibration to perceived fatigue
Brambilla et al. [38]	Systematic review of laboratory and industrial upper-limb assessments	EMG, kinematics, questionnaires, and other biomechanical tools	Identified heterogeneous protocols and a laboratory-to-workplace translation gap	Establishes the Industry 5.0 ergonomic context and need for multimodal, field-relevant validation
Kuber et al. [40]	Exoskeleton-assisted trunk-flexion tasks	Wearable EMG and IMU features with machine learning	Classified perceived back and leg fatigue levels	Demonstrates predictive sensor fusion in industrially relevant tasks; different body region and externally assisted task
Daniel et al. [41]	Review of dynamic-movement sEMG fatigue research	sEMG methods and experimental-design guidance	Synthesized confounders and validation requirements for dynamic fatigue studies	Directly supports the cautious interpretation of spectral shifts during changing posture and force
Coraggio et al. [29]	Repetitive upper-limb movements with sEMG fatigue analysis	Multiple linear spectral and amplitude-based fatigue indices	Demonstrated that algorithm performance depends on movement and fatigue-index definition	Supports task-specific comparison rather than assuming a universally superior spectral index
Oliveira et al. [14]	Review of unobtrusive home monitoring toward Healthcare 5.0	Camera, radar, mechanical, bioelectrical, optical, and multimodal sensing	Identified opportunities and validation gaps for continuous personalized monitoring	Extends the motivation to preventive and personalized health; did not assess haptic workload or sEMG fatigue
Present study	Repetitive force–feedback writing in virtual reality	Five-channel sEMG, embedded accelerometry, stylus force, and position	Compared MNF- and MDF-derived arm indices using association, influence, calibration, and scale-correspondence analyses	Adds a dynamic haptic task, five-muscle fusion, small-sample influence analysis, and held-out internal calibration

**Table 2:** Historical processing information and prospective replication specification. The right-hand column contains recommendations for future raw-signal replication only; none of those recommended filter, spectral-band, QC, or sensitivity settings were used to generate the archived participant-level results reported in this manuscript.

Element	Historical Analysis Record	Prospective Reproducible Specification
Sampling/segmentation	1000 Hz; 125-sample (0.125 s) window; 62-sample hop; 63 shared samples (50.4% overlap), consistent with the earlier report [31]	Retain these settings as the continuity analysis; repeat with 250 ms (4 Hz bins) and 500 ms (2 Hz bins) windows
Candidate windows	6450 per uninterrupted 400 s muscle record; 32,250 per participant; 645,000 participant×muscle candidate windows before QC	Report retained/6450 and percentage for every participant×muscle series, plus cohort median [IQR] and min–max
Taper/spectrum	Hann taper; 8 Hz DFT-bin spacing; one-sided periodogram; interpolated MDF	Retain the same taper, periodogram, and interpolated half-power MDF estimator
Band-pass filtering	Bessel filtering documented in the earlier peer-reviewed report [31]; historical order and cutoffs not stated	Recommended prospective primary analysis: 4th-order digital Butterworth 20–400 Hz, applied bidirectionally offline; recommended method-continuity sensitivity: 4th-order Bessel 20–400 Hz; recommended bandwidth sensitivity: 20–450 Hz
Power-line interference	Power-line removal documented; historical numerical implementation not retained	Inspect 60 Hz contamination for the Ottawa acquisition setting. Avoid routine notching of fatigue spectra; if residual line components are detected, remove fitted sinusoidal components at 60 Hz harmonics within the retained band and report each removed harmonic
Spectral limits	$\left[f_{L},f_{U}\right]$ documented symbolically; numerical historical limits not retained	Recommended prospective primary band: 20–400 Hz, with 20–450 Hz sensitivity analysis; report exact retained DFT-bin centers
Window quality control	Invalid windows were omitted; historical numerical thresholds and retained counts not retained	Hard reject non-finite samples, zero in-band power, or confirmed clipping/saturation. Flag, rather than automatically delete, RMS/total-power/line-contamination outliers with robust |z|>5 (MAD-based) for manual review and sensitivity analysis; no imputation
Temporal model/aggregation	Quadratic trajectory over normalized time; signed fitted start-to-end decline; equal five-muscle averaging	Retain the same model as the primary replication and report per-muscle fit quality, direction of change, and accepted-window count before aggregation
Code/audit trail	Original raw-signal preprocessing code not retained	Archive executable preprocessing, window-retention log, parameter file, and participant-level analysis code. The present revision includes code reproducing the leave-one-participant-out influence and LOOCV internal-calibration statistics from the retained participant-level arm-index table

**Table 3:** Principal notation used in the signal-processing and fatigue-index equations.

Symbol	Definition	Symbol	Definition
p,N	Participant index and number of participants (N=20)	i,K	Muscle index and number of muscles (K=5)
$n,N_{s}$	Sample index and recording length in samples	q,Q	Window index and number of complete windows
$f_{s}$	Sampling frequency (1000 Hz)	L,H	Window length and hop size in samples
$s_{p,i}\left[n\right]$	Raw sEMG sample	$x_{p,i}\left[n\right]$	Filtered, mean-removed sEMG sample
w[ℓ],U	Taper and taper-energy normalization	X[j],P[j]	Fourier coefficient and power-spectral density
$j,f_{j}$	Frequency-bin index and frequency in hertz	J	Set of bins retained for spectral analysis
$C\left[k\right],P^{\mathrm{tot}}$	Cumulative and total retained spectral power	$k^{*}$	First bin reaching half of total power
$z_{p,i,q}$	MNF or MDF value for one window	$\tau_{q}$	Window time normalized to [0, 1]
a,b,c	Quadratic curvature, linear, and intercept coefficients	$Q_{p,i}$	Set of windows passing quality control
$g\left(\tau \right),\tau^{*}$	Fitted trajectory derivative and interior turning point	$R_{p,i,z}^{2}$	Quadratic trajectory coefficient of determination
${\mathrm{FI}}_{p,z}^{\left(i\right)}$	Muscle-level spectral-decline index (%)	${\mathrm{FI}}_{p,z}^{\mathrm{arm}}$	Mean index across the five muscles (%)
$r_{p,h}$	Seven-point response to questionnaire item *h*	$n_{h,k},P_{h,k}$	Count and percentage of responses in category *k* for item *h*
$Y_{p}$	Archived participant-level 0–100 analysis criterion, reported separately from raw Q2	$e_{p,z}$	Difference between arm-level index *z* and $Y_{p}$

**Table 4:** Status of reviewer-requested robustness analyses and prospective raw-signal replication plan. “Conducted” denotes analyses that can be reproduced from the retained participant-level arm-index table. “Prospective only” denotes analyses that require participant-by-muscle/window, channel-level, QC-log, or participant-linked mechanical records that are not retained in the historical dataset.

Robustness Question	Status in This Revision	Analysis/Prospective Requirement
Participant influence	Conducted	Leave-one-participant-out recomputation of $r_{\mathrm{MDF},Y}$, $r_{\mathrm{MNF},Y}$, and Δr for all 20 deletions; results are reported in Section 4.6 and summarized in the inferential-hierarchy results table.
Internal calibration	Conducted	Leave-one-participant-out calibration of each arm index to archived $Y_{p}$; held-out MAE, RMSE, $R^{2}$, correlation, bias, limits, and maximum error are reported in the internal-calibration results table.
Spectral resolution	Prospective only	Repeat the raw-signal pipeline with 250 ms and 500 ms windows (4 and 2 Hz native bin spacing) and compare participant-level FI values, rank stability, and the MDF-MNF correlation-difference ordering.
Trajectory model	Prospective only	Recompute muscle-level trajectories with a linear fit and a robust smooth trend; report fitted start/end values, adjusted $R^{2}$, normalized residual error, monotonicity, and interior turning points.
Muscle aggregation	Prospective only	Recompute five leave-one-muscle-out arm indices; add activation-weighted sensitivity only when independently normalized RMS activation is available; no weight may be optimized against $Y_{p}$.
Task mechanics	Prospective only	Derive participant-level mean force, force SD/CV, movement amplitude/path length, and RMS acceleration and test parsimonious mechanically adjusted FI–criterion associations.
Channel plausibility	Prospective only	Report fitted initial *c* and final a+b+c frequency, FI denominator, accepted-window count, clipping/line-noise flags, and nonpositive or out-of-band fitted endpoints for every participant×muscle record.
Muscle-level consistency	Prospective only	For each muscle report MNF/MDF decline distribution, positive-FI proportion, fit-quality distribution, monotonicity/turning-point counts, and retained-window summaries.

**Table 5:** Specification of the post-task arm-fatigue questionnaire item and the separate archived participant-level analysis criterion.

Element	Specification
Raw questionnaire item	Q2: “Please indicate your current arm fatigue level?”; seven categories; 1 = “not at all,” 7 = “completely”
Recorded aggregate Q2 distribution	5: n=2; 6: n=9; 7: n=9; mean 6.35/7
Direct 0–100 rescaling of raw Q2	$C_{p}=100\left(r_{p,2}-1\right)/6$ when the participant-level raw category is known; aggregate mean corresponding to the recorded Q2 counts = 89.17/100
Archived participant-level analysis criterion	$Y_{p}$, 0–100; range 25–90; mean 56.95; used in the legacy participant-level comparison
Analytical separation	Raw Q2 is reported descriptively; $Y_{p}$ is used only for the archived-scale association, calibration, and scale-correspondence analyses

**Table 6:** Numerical provenance diagnostic: recorded raw Q2 counts versus nearest-level projection of archived $Y_{p}$. Exact midpoint ties at $Y_{p}=25$ and 75 are assigned to the lower admissible category for this diagnostic. The mismatch confirms that the projection is not a reconstruction of the recorded raw responses.

Q2 Category	Raw Q2 *n*	Raw Q2 (%)	Nearest-$Y_{p}$ *n*	Projected (%)
1	0	0	0	0
2	0	0	1	5
3	0	0	3	15
4	0	0	7	35
5	2	10	6	30
6	9	45	3	15
7	9	45	0	0

**Table 7:** Study and protocol characteristics.

Characteristic	Value
Participants	20 (12 men, 8 women)
Age range	25–59 years
Recorded task duration	400 s
Haptic interface	Geomagic Touch
sEMG sampling frequency	1000 Hz
Monitored muscles	Five upper-limb muscles
Post-task scale	Six items, 1–7 response scale

**Table 8:** Participant-level arm-fatigue indices and archived participant-level analysis criterion $Y_{p}$, expressed on the 0–100 analysis scale. Raw Q2 questionnaire outcomes are reported separately. The largest MNF (93.18%) and MDF (81.87%) values are participant-level maxima for F8, not cohort averages.

Participant	${\mathrm{FI}}_{\mathrm{MNF}}$ (%)	${\mathrm{FI}}_{\mathrm{MDF}}$ (%)	$Y_{p}$ (%)
M1	43.22	35.00	40
M2	50.21	37.00	60
M3	49.12	22.00	30
M4	42.10	59.00	60
M5	70.10	75.30	90
M6	40.10	45.66	50
M7	51.74	21.00	25
M8	42.10	35.00	40
M9	53.20	39.00	47
M10	67.10	59.00	60
M11	45.22	50.00	58
M12	51.50	42.30	44
F1	60.30	45.60	56
F2	70.90	66.60	65
F3	49.40	39.80	44
F4	69.91	65.90	75
F5	80.45	73.10	80
F6	50.79	48.10	50
F7	70.49	71.10	75
F8	93.18	81.87	90

**Table 9:** Exploratory leave-one-participant-out internal calibration of each arm index to the archived $Y_{p}$ analysis scale. The full-sample equation is shown for descriptive calibration; performance metrics are calculated exclusively from held-out predictions. This analysis is not external validation and is not an agreement analysis with raw Q2.

Index	Full-Sample Calibration	MAE	RMSE	$R^{2}$	*r*	Bias	95% LoA	Max |e|
MDF	Y=6.939+0.988FI	4.38	5.87	0.891	0.944	−0.06	−11.87, 11.75	17.97
MNF	Y=0.438+0.982FI	9.24	12.02	0.542	0.739	−0.10	−24.28, 24.08	27.87

Errors are percentage points on the archived $Y_{p}$ scale. LOOCV, leave-one-participant-out cross-validation. The analysis evaluates internal calibration only; it does not establish interchangeability with raw Q2 or external predictive validity.

**Table 10:** Exploratory association and uncalibrated scale correspondence of the two arm-fatigue indices relative to the archived $Y_{p}$ analysis criterion. These raw difference metrics are descriptive scale-correspondence diagnostics, not evidence of method agreement or interchangeability. Raw Q2 questionnaire responses are summarized separately, and the seven-level discretization stress test is not a reconstruction of raw Q2.

Metric	MNF Index	MDF Index
Pearson *r* (95% CI)	0.783 (0.521–0.910)	0.954 (0.885–0.982)
$R^{2}$	0.613	0.910
Spearman ρ	0.677	0.933
** Seven-level discretization stress test **		
Pearson *r*	0.722	0.908
Spearman ρ	0.664	0.892
Ordering retained after quantization: MDF > MNF for both association measures.		
MAE, percentage points	8.59	6.49
RMSE, percentage points	11.08	8.28
MAPE, % of archived score	19.18	11.92
Bias, percentage points	0.61	−6.33
95% conventional limits of agreement	−21.63 to 22.85	−17.05 to 4.39
Empirical 2.5th–97.5th difference interval	−18.95 to 23.12	−19.06 to 0.37
Bias, discretization stress test	1.72	−5.22
95% LoA, discretization stress test	−23.04 to 26.49	−20.29 to 9.86

MAE, mean absolute error; RMSE, root-mean-square error; MAPE, mean absolute percentage error; LoA, limits of agreement. MAE, RMSE, bias, and LoA are expressed in percentage points. MAPE is a relative percent-of-score quantity and is reported descriptively because its denominator is a bounded 0–100 score. Differences were calculated as objective index minus archived *γ*_p_ value. These uncalibrated values describe scale correspondence, not interchangeability with raw Q2. The separate LOOCV analysis in Table 9 evaluates held-out internal calibration.

**Table 11:** Inferential hierarchy, multiplicity handling, and resampling sensitivity analyses.

Analysis Role	Result and Interpretation
Planned within-sample comparison (exploratory)	Dependent correlation difference: Steiger p=0.0017, Holm-adjusted p=0.0035; bootstrap 95% CI for Δr, 0.041–0.373
Randomization sensitivity	Paired MDF/MNF label-swap test: p=0.082; stronger exchangeability null not rejected
Interpretive rule	MDF is described as the more promising task-specific candidate for prospective validation because it has the higher point estimates and stable participant-deletion ordering; the non-significant label-swap test precludes a claim of universal superiority.
Formal secondary comparison	Paired absolute-error difference: p=0.274, Holm-adjusted p=0.274; exact sign-flip p=0.281
Descriptive/sensitivity outputs	Individual correlations, Spearman ρ, MAE, RMSE, MAPE, bias, LoA, diagnostics, questionnaire distributions, and seven-level discretization results were not treated as separate confirmatory tests

## Data Availability

The participant-level data are not publicly available because they arise from human-participant research governed by the approved ethics protocol and institutional data-protection requirements. The revised submission package includes a participant-level analysis script that reproduces the leave-one-participant-out influence and LOOCV internal-calibration results from the values reported in Table 8. The raw-signal robustness analyses listed as “prospective only” in Table 4 were not conducted. The original historical raw-signal preprocessing code, complete QC logs, participant-by-muscle/window matrix, fitted channel-level endpoints, and participant-linked mechanical summaries were not retained. Requests for access to de-identified source data or recoverable historical analysis materials may be directed to the corresponding author and will be considered subject to participant consent, Research Ethics Board requirements, and University of Ottawa data-governance approval.

## Abbreviations

Abbreviations used in this manuscript:

EMG	Electromyography
sEMG	Surface electromyography
MNF	Mean frequency
MDF	Median frequency
MVC	Maximum voluntary contraction
PSD	Power-spectral density
VR	Virtual reality
MAE	Mean absolute error
RMSE	Root-mean-square error
MAPE	Mean absolute percentage error
